# Sports Preparticipation Evaluation for Healthy Adults: A Consensus-Based German Guideline

**DOI:** 10.1007/s40279-025-02230-5

**Published:** 2025-07-03

**Authors:** Christine Joisten, Anja Hirschmüller, Pascal Bauer, Erika Baum, Meinolf Behrens, Susanne Berrisch-Rahmel, Gregor Berrsche, Anja Carlsohn, Michael Cassel, Justus DeZeuuw, Gesine Dörr, Michael Dreher, Frank Edelmann, Katrin Esefeld, Michael Freitag, Mathias Grebe, Casper Grim, Pia Janßen, Rolf Kaiser, Thomas Katlun, Maximilian Köppel, Charlotte Kreutz, Karsten Krüger, Christoph Lutter, Frank Mayer, Othmar Moser, Andreas Nieß, Hans-Georg Predel, Stefan Peters, Petra Platen, Dorothea Portius, Claus Reinsberger, Nils Reiss, Kai Röcker, Thomas Schmidt, Arno Schmidt-Trucksäss, Holger Schmitt, Thomas Schramm, Christian Sturm, Hans Vater, Alina Weise, Burkhard Weisser, Götz Welsch, Andreas Winkelmann, Alfred Wirth, Bernd Wolfarth, Käthe Goossen

**Affiliations:** 1 Institute of Movement and Neurosciences, German Sport University Cologne, Am Sportpark Müngersdorf 6, 50933 Cologne, Germany; 2 German Society of Sports Medicine and Prevention, Frankfurt am Main, Germany; 3 Altius Swiss Sportmed Center Rheinfelden, Habich-Dietschy-Strasse 5a, 4310 Rheinfelden, Switzerland; 4 Department of Orthopedics and Trauma Surgery, Medical Center, Albert-Ludwigs-University of Freiburg, Freiburg, Germany; 5 Department of Cardiology and Angiology, Justus Liebig University, Giessen, Germany; 6 German Society for Cardiology-Cardiovascular Research (DGK), Düsseldorf, Germany; 7 Department of General Practice, University of Marburg, Marburg, Germany; 8 German Society for General Practice and Family Medicine (DEGAM), Frankfurt am Main, Germany; 9 Diabetes Centre Minden, Minden, Germany; 10 German Diabetes Society (DDG), Working Group Diabetes, Sport, and Exercise Germany, Berlin, Germany; 11 KardioPro, Practice for Internal Medicine, Cardiology, Sports Medicine, and Sports Cardiology, Düsseldorf, Germany; 12 Centre for Orthopaedics and Sports Trauma Surgery, Atos Clinic Heidelberg, Heidelberg, Germany; 13 Society for Orthopaedic-Traumatological Sports Medicine (GOTS), Jena, Germany; 14 Department of Nutrition and Home Economics, University of Applied Sciences Hamburg, Hamburg, Germany; 15 Faculty of Health Sciences, University of Potsdam, Potsdam, Brandenburg, Germany; 16 Healthy Heart MVZ in Cologne, Cologne, Germany; 17 German Society for Pneumology and Respiratory Medicine (DGP), Berlin, Germany; 18 Alexianer St. Josefs Hospital Potsdam-Sanssouci, Potsdam, Germany; 19 German Society for Angiology-Society for Vascular Medicine (DGA), Leipzig, Germany; 20 Department of Pneumology and Intensive Care Medicine, University Hospital RWTH Aachen, Aachen, Germany; 21 Department of Internal Medicine and Cardiology, Campus Virchow Hospital, Charité University Medicine Berlin, Berlin, Germany; 22 German Society for Internal Medicine (DGIM), Berlin, Germany; 23 Department of Prevention and Sports Medicine, University Hospital Klinikum Rechts Der Isar, Technical University of Munich, Munich, Germany; 24 Department of General Medicine, Department of Health Services Research, Carl Von Ossietzky University Oldenburg, Oldenburg, Germany; 25 Centre of Cardiac and Vascular Diseases, Marburg, Germany; 26 Centre for Musculoskeletal Surgery Osnabrück, Osnabrück Hospital, Osnabrück, Germany; 27 German Olympic Sports Confederation (DOSB), Frankfurt am Main, Germany; 28 Department of Sports Medicine, University Hospital of Tübingen, Tübingen, Germany; 29 Clinic for Internal Medicine I, Cardiology, Angiology, Diabetology and Sports Medicine, Hanse and University City of Rostock, Rostock, Germany; 30 German Disabled Sports Association and National Paralympic Committee E.V. (DBS), Frechen, Germany; 31 Katlun Eye Clinic, Heidelberg, Germany; 32 Working Group Oncological Sports and Exercise Therapy, National Centre for Tumour Diseases Heidelberg, Heidelberg, Germany; 33 German Association for Health-Related Physical Activity and Exercise Therapy (DVGS), Hürth, Germany; 34 Department of Exercise Physiology and Sports Therapy, Institute of Sports Science, Giessen, Germany; 35 Department of Orthopaedics, Rostock University Medical Center, Rostock, Germany; 36 University of Potsdam, Centre of Sports Medicine, University Outpatient Clinic, Potsdam, Germany; 37 Exercise Physiology and Metabolism (Sports Medicine), Bayreuth Centre of Sports Science, University of Bayreuth, Bayreuth, Germany; 38 German Sport University Cologne, Institute of Cardiology and Sports Medicine, Cologne, Germany; 39 German Hypertension League (DHL), Heidelberg, Germany; 40 Department of Human Sciences, Institute of Sport Science, Bundeswehr University Munich, Neubiberg, Germany; 41 Department of Sports Medicine and Sports Nutrition, Faculty of Sports Science, Ruhr University Bochum, Bochum, Germany; 42 Martin Luther University Halle-Wittenberg, Institute of Agricultural and Nutritional Sciences, Halle, Germany; 43 German Obesity Society (DAG), Berlin, Germany; 44 Institute of Sports Medicine, Paderborn University, Paderborn, Germany; 45 Schüchtermann Clinic Bad Rothenfelde, Bad Rothenfelde, Germany; 46 German Society for Prevention and Rehabilitation of Cardiovascular Diseases (DGPR), Potsdam, Germany; 47 Institute for Applied Health Promotion and Exercise (IFAG), Furtwangen University, Furtwangen, Germany; 48 Sport and Exercise Medicine, Department of Sport, Exercise and Health, University of Basel, Basel, Switzerland; 49 Cardiology Rodenkirchen, Cologne, Germany; 50 Department of Rehabilitation and Sports Medicine, Hannover Medical School, Hannover, Germany; 51 German Society for Physical and Rehabilitative Medicine (DGPRM), Munich, Germany; 52 Prof Vater & Colleagues, Bad Wildungen, Germany; 53 Witten/Herdecke University, Institute for Research in Operative Medicine (IFOM), Cologne, Germany; 54 Institute for Sports Science, Christian-Albrechts-University Kiel, Kiel, Germany; 55 Athleticum, Department of Sports Medicine and Department of Trauma and Orthopedic Surgery at University Medical Center Hamburg-Eppendorf (UKE), Hamburg, Germany; 56 German Society for Orthopaedics and Trauma Surgery (DGOU), Berlin, Germany; 57 Department of Orthopaedics and Trauma Surgery, Musculoskeletal University Center Munich (MUM), LMU University Hospital, LMU Munich, Munich, Germany; 58 Teutoburger Wald Clinic, Bad Rothenfelde, Germany; 59 Department of Sports Medicine, Humboldt University and Charité University School of Medicine, Berlin, Germany

## Abstract

The benefits of physical activity are undisputed. However, adverse events can occur in rare cases, particularly during high-intensity or prolonged exercise. During physical activity, at-risk patients can experience major cardiac events, whereas adverse events affecting the musculoskeletal system are more common but less severe. A sports preparticipation evaluation (PPE) for apparently healthy adults is designed to detect at-risk individuals and prevent potentially fatal events. This guideline for conducting PPEs was developed by consensus among 16 medical societies and sports associations and is based on previously published guidelines and consensus papers. Sports medicine physicians and potential participants were also surveyed to assess the recommendations’ content, feasibility, and implementation. On the basis of the 20 recommendations developed and agreed upon by the abovementioned entities, PPE comprises individuals’ personal, family, and sports histories, as well as a physical examination. The need for additional examinations (e.g., laboratory parameters, echocardiograms, or stress tests) is determined on the basis of the PPE findings. This approach’s feasibility in various regions, including resource-limited settings, and the extent to which it prevents adverse or potentially fatal events, should be examined in future research.

**Supplementary Information:**

The online version contains supplementary material available at 10.1007/s40279-025-02230-5.

## Key Points

**Table:** 

This new consensus-based sports preparticipation evaluation (PPE) guideline is intended for healthy adults who plan to start exercising regularly or at higher intensities.
The PPE serves to screen for individuals at risk of fatal events while exercising.
These PPE guidelines are based mainly on individuals’ medical history and physical examination results. Further examinations are recommended only if abnormalities are detected.
This PPE can also provide a foundation for individual training recommendations, increasing an individual’s motivation to pursue an active lifestyle.
National and international register studies are required to assess the benefits of this PPE and reevaluate our recommendations.

## Introduction

Physical activity undisputedly offers widespread benefits across all age groups and sexes, including preventing and treating chronic diseases [1, 2]. The World Health Organization currently recommends 150–300 min of moderate-intensity exercise at least 5 days per week or 75–150 min of high-intensity exercise at least 3 days per week; the more, the better [1, 2]. However, individuals with underlying or undiagnosed medical conditions, especially those who engage in sudden, high-intensity exercise, may face health risks, including musculoskeletal injuries and, more rarely, serious cardiac events [3–5]. This phenomenon, often called the “exercise paradox”, highlights that while exercise reduces long-term cardiovascular risk, it can also trigger acute cardiac events in vulnerable individuals [6]. A sports preparticipation evaluation (PPE) is recommended to mitigate such risks by identifying at-risk individuals and providing guidance on safe physical activity [4].

To date, most international recommendations for PPEs have focused on elite athletes and are primarily based on expert consensus derived from organized competitive sports, such as the current European Federation of Sports Medicine Associations (EFSMA) guideline on preparticipation medical evaluation for elite athletes [7]. The Italian Working Group has recently updated the Italian Cardiological Guidelines (COCIS) for Competitive Sport Eligibility in athletes with heart disease [8]. Only few guidelines have specifically addressed recreational athletes or individuals who are new or returning to exercise [9]. In this context, the American College of Sports Medicine (ACSM) proposed a two-stage screening process before the start of moderate or intense training [10]. The European Association for Cardiovascular Prevention and Rehabilitation (EACPR) [11] has also introduced screening guidelines for middle-aged and older adults before exercise that vary according to the individual’s cardiac risk profile and the intended level of physical activity. Ermolao et al. [12] compared the EACPR and ACSM guidelines [13, 14], finding that 49% of cardiovascular conditions were missed by the EACPR guidelines, 29% by the older ACSM guidelines, and 50% by the new ACSM guidelines.

Therefore, the most effective and practical methods of evaluating sports-related risks in apparently healthy adults (aged ≥ 18 years) who are starting or resuming intense physical activity remain to be identified. To address this need, a consensus-based guideline was developed under the auspices of the German Society for Sports Medicine and Prevention (Deutsche Gesellschaft für Sportmedizin und Prävention [DGSP]) [15]. This guideline is based on current international recommendations and is aimed at healthy adults, including individuals recovering from conditions such as cancer, joint injuries, or joint surgeries—not elite athletes. The PPE should be performed by specialized sports physicians who are adequately trained in the cross-disciplinary nature of the evaluation (for specialist medical training see European Union [EU] Article 26 of Directive 2005/36/EC [16] or course-based medical training in sports medicine, Germany [17]). The PPE may also serve as a baseline for exercise-related counseling, applying the frequency, intensity, time, type, volume, and progression (FITT-VP) principle. In addition, the individual’s cardiorespiratory and muscular fitness should be assessed owing to their protective nature against noncommunicable diseases [18, 19].

The aim of this paper was to provide an overview of the consensus-based guidance that was published on the Guidance Manual of the German Association of Scientific Medical Societies (Arbeitsgemeinschaft der Wissenschaftlichen Medizinischen Fachgesellschaften [AWMF]) register [15] and to contextualize the recommendations for an international audience. In addition, we summarized the approach and methods employed and provided further detail of the screening algorithm, background considerations underlying each recommendation, resp. medical history and examination forms.

## Methods

**Fig. 1:**
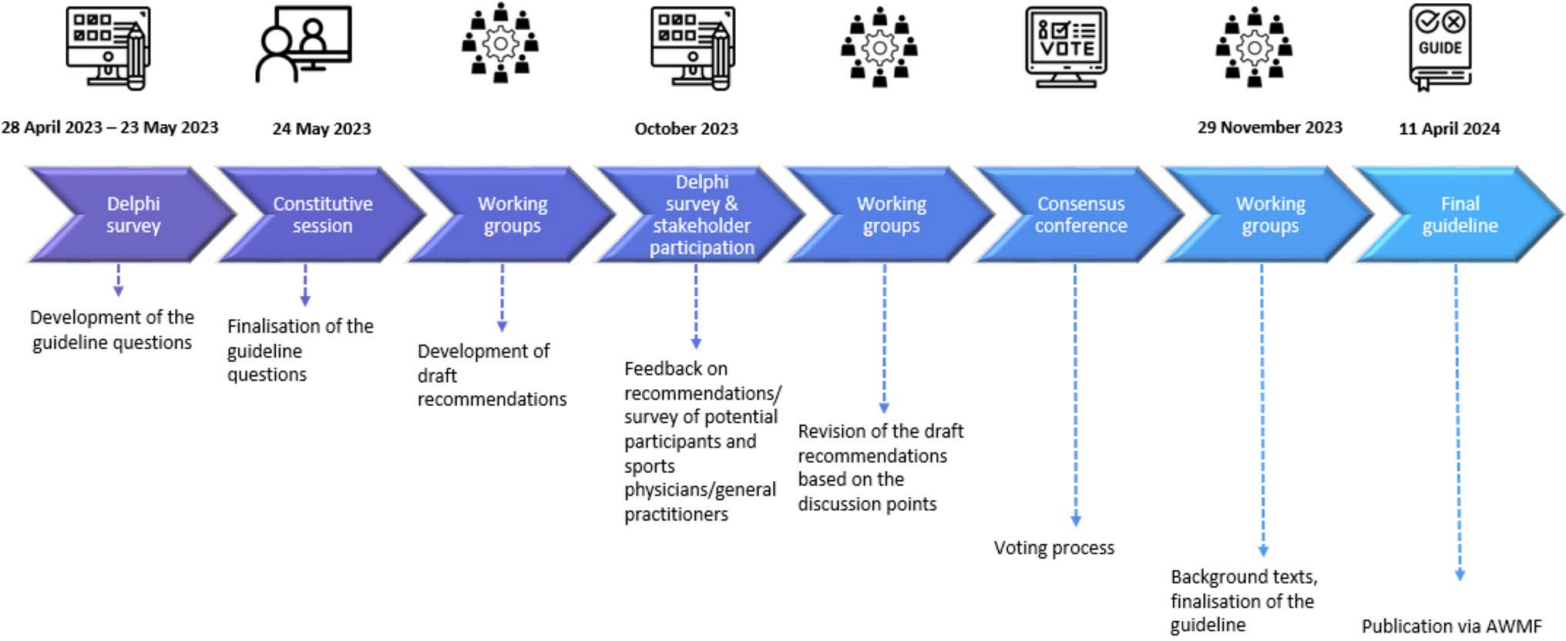
The guideline development process based on the Guidance Manual of the German Association of Scientific Medical Societies (Arbeitsgemeinschaft der Wissenschaftlichen Medizinischen Fachgesellschaften [AWMF]) [20], with timelines

The DGSP, a nonprofit specialty organization, commissioned this consensus-based guideline. It was developed on the basis of the Guidance Manual of the German Association of Scientific Medical Societies (Arbeitsgemeinschaft der Wissenschaftlichen Medizinischen Fachgesellschaften [AWMF]) and followed the process outlined in Fig. 1.

### Guideline Classification

German clinical practice guidelines are developed with a classification system (S-classification) that describes how much systematic methodology is applied [20]. The guideline classes range from S1 (recommendations by expert groups, developed using informal consensus) to S2e (evidence-based guidelines without formal consensus methods) and S2k (formal consensus-based guidelines without systematic evidence reviews) to S3 (formal consensus-based guidelines with systematic evidence reviews). The developing organization chooses the class according to how much effort is appropriate and feasible.

The DGSP was responsible for the guideline development and selected class S2k. Therefore, the development of this guideline involved a formal, structured consensus process within a representative committee but no systematic review of the evidence. We suspected that comparative studies could not adequately address many of the clinical questions for a guideline on sports PPEs (see Sect. 2.4). Therefore, the development of an evidence-based guideline would be neither feasible nor resource-efficient at this stage.

### Stakeholder Involvement

All relevant professional groups, including specialist societies and organizations, appointed delegates to the Guideline Development Group (Box 1). The DGSP’s executive and scientific advisory boards nominated a panel of 14 experts to support the development of recommendations in an advisory capacity; panel members were selected for their expertise in various specialist areas (e.g., sports cardiology, orthopedics, ophthalmology, and neurology).
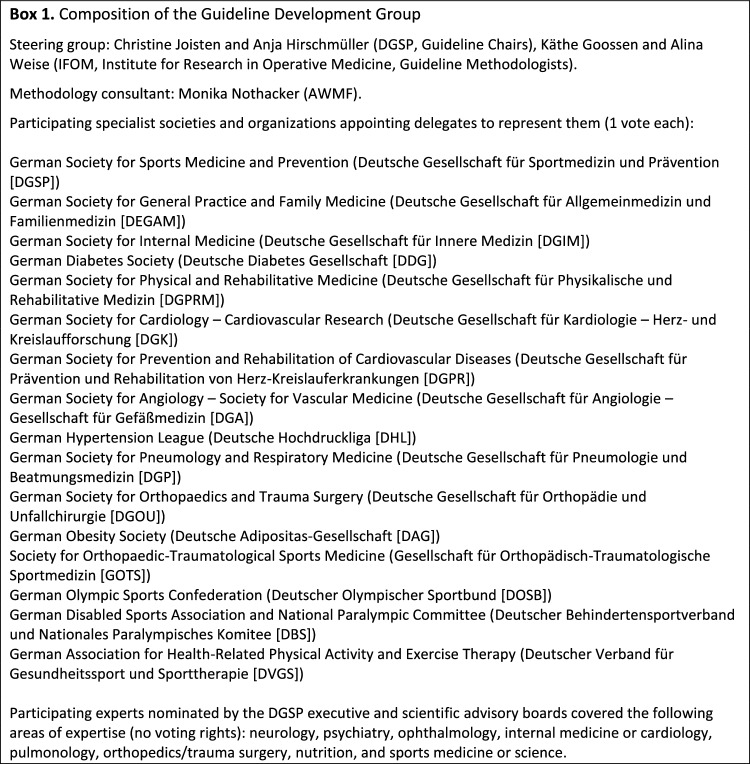


The views and preferences of the target population (i.e., potential participants) and the target users of the guideline (i.e., sports physicians and general practitioners) were collected with two online surveys [21]. The surveys addressed the acceptability and feasibility of the PPE, and respondents were asked to comment directly on the draft recommendations. Their feedback informed discussions about the final recommendations and remarks on views and preferences that were added to the complete guideline text.

### Management of Competing Interests

All authors of the guideline disclosed any direct, financial, or indirect interests using an online form. A peer group assessed the relevance and level of conflict indicated before the consensus conference (Supplementary Material 1).

### Systematic Review of Existing Guidelines and Recommendations

**Fig. 2:**
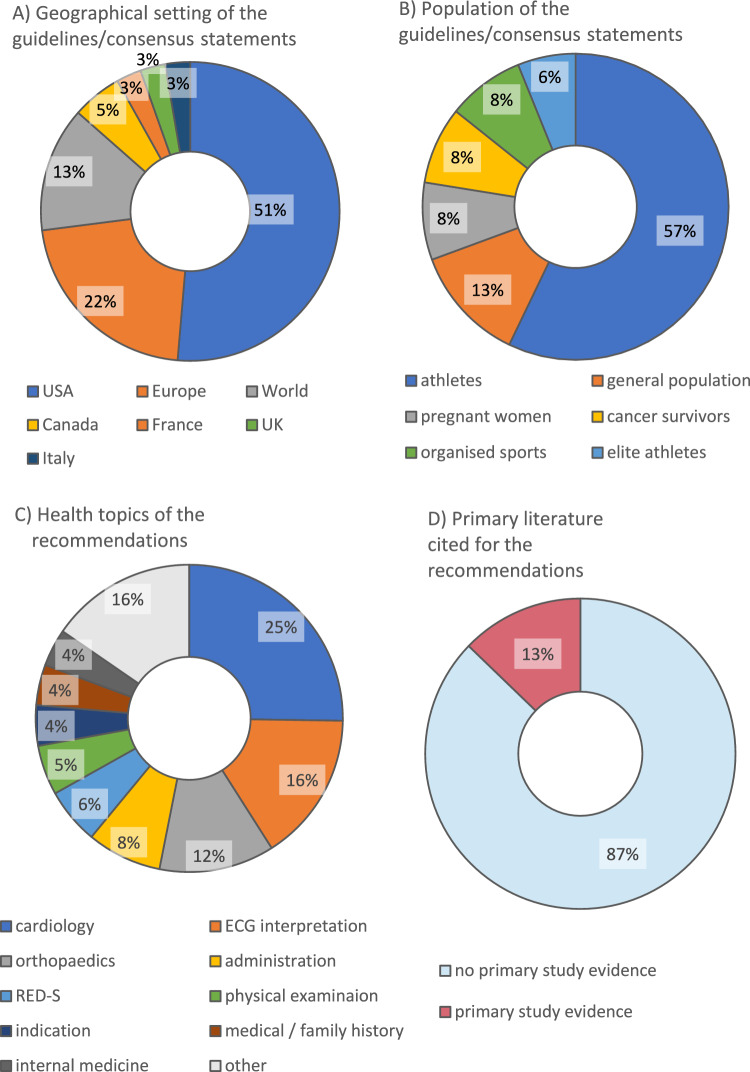
A systematic review of guidelines and consensus statements. The characteristics of the 35 included documents, sorted by **A** geography and **B** population, and the characteristics of the recommendations made within them, sorted by **C** health topic and **D** link to primary study evidence (*n* = 305 recommendations citing 55 primary studies) [9]. *ECG* electrocardiogram, *RED-S* relative energy deficit in sport

In preparation for guideline development, we systematically reviewed the evidence- or consensus-based recommendations for PPEs published since 2012 [9]. This review was intended to identify potential guideline questions and determine the availability of primary study evidence to support the PPE recommendations. It resulted in 35 guidelines and consensus statements identified from developed countries worldwide (Fig. 2). These mainly targeted athletes or participants in organized sports but some targeted the general population or specific subgroups. A total of 305 recommendations were made over various topics. Most recommendations (87%) did not cite evidence from primary studies.

### Guideline Questions

On the basis of the systematic review (Sect. 2.4), the steering group proposed guideline questions [9] that were revised, discussed, and finalized in an online Delphi process, including a survey and consensus meeting [15].

### Evidence Base

The systematic review (Sect. 2.4) was used to obtain evidence from primary studies as follows. Whenever the 35 selected guidelines and consensus documents (Supplementary Material 2) directly linked their recommendations to primary studies, these were used as evidence for our guideline (55 studies; Fig. 2D). However, most included documents were consensus-based and did not refer to primary studies as evidence. Therefore, the evidence base was supplemented with comparative studies of PPEs that were known to the authors of this German guideline or recommended for consideration by the participating experts. The German S2k class of guidelines does not generally entail a full systematic review of primary studies, and no such review was conducted (see Sect. 2.1 for the rationale).

Each guideline was appraised using Domains 3 (“Rigour of Development”) and 6 (“Editorial Independence”) of the Appraisal of Guidelines for Research and Evaluation-II (AGREE-II) [22, 23]. Each primary study was assigned a level of evidence [24].

### Development of the Recommendations

The delegates and experts formed three working groups for internal medicine, orthopedics, and other topics. Each working group reviewed the relevant primary evidence and prepared draft recommendations on the basis of the guideline questions. They reviewed the results of the stakeholder surveys, revised the recommendations, and wrote background text explaining the rationale for each recommendation.

### Strength of the Recommendations

The Guideline Development Group determined the strength of recommendations through a formal consensus procedure. Symbols and specific wording were used to express the grade of recommendation as defined in the AWMF Guidance Manual (Table 1) [20]. This table also indicates the corresponding class under the American College of Cardiology (ACC)/American Heart Association (AHA) Clinical Practice Guideline Recommendation Classification System that is widely used by organizations such as the European Society of Cardiology (ESC) [25]. The grading corresponds to the degree of certainty that the observed benefit of an intervention outweighs its potential harm and that the positive effects are relevant to the guideline population. When grading the recommendations, we considered the balance of benefit to harm, benefit to estimated cost, confidence in the evidence (where available), potential participants’ survey-reported views and preferences, sports physicians’ survey-reported perceptions of feasibility, and the Guideline Development Group’s clinical expertise.

**Table 1:** Grades of recommendations and classifications of consensus strength

Grade of recommendation	Corresponding ACC/AHA class [25]	Symbol, wording	Definition
Strong recommendation	Class I (strong) or class III: harm (strong)	⇑⇑ verb in the imperative tense	A net benefit is expected for most individuals, and no relevant groups who would not benefit are known
Moderate recommendation	Class IIa (moderate) or class III: no benefit (moderate)	⇑ modal verb “should”	A net benefit is expected for many individuals, but relevant groups who would not benefit are known Or: The expected benefit is estimated to be small
Weak recommendation	Class IIb (weak)	⇔ modal verb “can”	Uncertain net benefit Or: A net benefit is expected only for specific individuals, or the intervention is not recommended as a standard of care

Consensus strength	Approval rate^a^	Consequence
Strong consensus	≥ 95% of eligible votes^b^	The recommendation was approved
Moderate consensus	> 75–95% of eligible votes^b^	The recommendation was approved, and diverging views were documented alongside the recommendation or in the background text
Majority agreement	50–75% of eligible votes^b^	The recommendation was revised to account for diverging views, followed by a repeat vote
No majority agreement	< 50% of eligible votes^b^	The recommendation was rejected

^a^Recommendation may be for or against an intervention

^b^Eligible votes consisted of one vote per specialist society/organization, excluding delegates with moderate or strong conflicts of interest

### Consensus Procedure

Consensus was reached via a two-step process. The first step was an online Delphi survey that asked the Guideline Development Group to comment on and vote for the selected recommendations. This step was applied only to recommendations with a low expected need for discussion (i.e., recommendations that achieved consensus among all working group members and had received few comments in the acceptability and feasibility surveys). Recommendations were approved if they reached a strong consensus (Table 1) and received only editorial remarks. The second step was a structured consensus conference, which was held on 29 November 2023 as a Zoom-based web conference and moderated by the guideline methodologist with the support of an external neutral methodology consultant trained in structured consensus-seeking methods. At this conference, participants discussed, modified (if necessary), voted on, and endorsed recommendations and their corresponding grades. Each specialist society and organization had one vote. Voting was anonymous. The consensus strength (Table 1) was documented alongside the recommendations, and no recommendations with an approval rate of ≤ 75% were included, so all recommendations were approved with a strong or moderate consensus.

If individual specialist societies and organizations disagreed with the wording or grade of a recommendation, they were allowed to express their views by submitting an alternative recommendation wording or grading with a justification. Documentation of diverging views still led to the acceptance of any recommendation with an approval rate of > 75% (Table 1, moderate consensus).

### Peer Review

In the peer consultation phase, the guideline’s key recommendations and background texts were submitted to all participating societies and organizations for review by their boards and executive committees. A structured comment form was used to record their suggestions for changes and justifications. The results were then discussed by the guideline coordinators and incorporated into the guideline text.

### Overall Approval and Updating

The guideline was formally approved by the executive boards of all participating societies and organizations and was accepted for publication by the AWMF on 11 April 2024 [15]. The guideline is set to be updated in March 2029. If new scientific findings arise that call its recommendations into question, updates will be made earlier.

### Editorial Independence

The guideline development process was unfunded; all members of the working groups were unpaid volunteers. The methodology team received funding from the nonprofit lead specialist society (DGSP) to prepare the synopsis, implement the surveys, and develop the guideline methodology. They received no additional financial support. Most importantly, no support from industry or for-profit funding organizations was used.

## Recommendations

The following recommendations for PPEs for healthy adults who intend to start or return to intense physical activities were established through the consensus process described above.

### Population and General Advice

#### Recommendation 1

**Table:** 

A PPE should be offered to adults practicing or intending to start sports	
*Recommendation grade*: moderate	⇑
*Strength of consensus*: 85%* (moderate)	
*Justification*: The available outcome data are insufficient to offer every adult an appropriate examination, especially for light- to moderate-intensity sporting activities. The idea that every sporting activity entails increased risks should be avoided. However, the Guideline Development Group assumes that the benefits of an examination outweigh the risks because it can prevent exercise-related adverse events. In addition, the examination results can be used to motivate individuals to adopt active lifestyles	
* DEGAM diverging view: A PPE can be offered to adults practicing or intending to start sports There is insufficient evidence about this recommendation’s benefits and harms. While the Guideline Development Group assumed a main benefit, in the opinion of the DEGAM, the potential harm and the impact on care were insufficiently recognized. First, such an offer would entail a considerable commitment of human resources, which are currently—and will increasingly be—lacking, worsening care for the rest of the population, or overburdening existing healthcare staff. Second, a “should” (moderate) recommendation means considerable uncertainty for those involved: Should sports be started and continued only after a comprehensive preventive medical check-up? Such a recommendation could discourage individuals from starting or maintaining physical activity. After all, a repeat examination is recommended every 1–5 years. In addition, whether a specific fitness level for sports can be certified on the sole basis of such an examination, given insufficient evidence, remains in question, which the DEGAM clearly rejects	

Health checks are an essential element of preventive measures in Germany, where individuals with statutory health insurance are entitled to a one-off health check-up between the ages of 18 and 35 years. From 35 years of age, a check-up can be conducted once every 3 years. The aim is to identify common diseases, such as cardiovascular diseases and type 2 diabetes mellitus, as well as their risk factors, early. Screening measures are performed on individuals without symptoms of the screened diseases. However, not all screening measures are inherently useful [26]. The potential harm that can arise from false-positive findings, overdiagnosis, and pathologization outweigh the potential benefits. In addition to the consequences for the individual being examined, avoidable costs—both direct and indirect—are incurred.

These aspects of general health check-ups also apply to PPEs. Because previous international recommendations for PPEs have been predominantly based on expert consensuses derived from organized competitive sports, few studies have specifically considered recreational athletes [9]. For such athletes, the ACSM recommends a two-stage screening process before participation in moderate or intense training programs [10]. On the basis of the ACSM screening algorithm, whether medical clearance is required before an exercise program is determined first; if any abnormalities are detected, medical clearance should be recommended. However, the manner of clearance should be left to physicians’ clinical judgment and discretion.

Price et al. [27] showed that applying the updated ACSM guidelines in sports medicine screening (tenth edition) reduced the number of medical referrals by around one third. In the context of long-distance running races, Schwellnus et al. [28] confirmed that a screening program was feasible and that fewer medical consultations were required overall, and serious or life-threatening events occurred less frequently after its implementation. They also showed that the number needed to treat (NNT) was 394 for all participating runners and as high as 177 for longer-distance runners (56 km). To prevent one serious (life-threatening/death) medical event, the NNT was 2670. In the Italian screening program, 2.0% of apparently healthy athletes were found to have various conditions, including coronary abnormalities, mitral valve prolapses, cardiac arrhythmias, bronchial asthma, and visual impairments [29].

#### Recommendation 2

**Table:** 

A PPE should be conducted when starting an intense sport or exercise program	
*Recommendation grade:* moderate	⇑
*Strength of consensus*: 100% (strong)	
*Justification:* The available data are insufficient to link sports medicine screening to a specific degree of intensity or volume because subjective assessment findings can vary between individuals. In principle, however, physical activity at higher intensities defined by metabolic equivalents (METs) is assumed to be associated with greater risk, particularly for sedentary individuals and/or those with preexisting conditions	

In the USA, a PPE is recommended before increasing the volume or intensity of physical activity [30]. The most common method of objectively quantifying intensity is the metabolic equivalent (MET), which is a ratio of the metabolic cost induced by different types of exercise and intensity compared with the metabolic cost of sitting quietly [31]: light, < 3.0 MET; moderate, 3.0–5.9 MET; and vigorous, ≥ 6.0 MET. However, the Canadian Academy of Sport and Exercise Medicine does not consider a medical examination necessary before light to moderate physical activity; this position is endorsed internationally by many sports medicine organizations [32]. Three decades ago, Mittleman et al. [33] showed that intense physical activity increases the risk of cardiovascular events in previously inactive individuals. By surveying 1228 individuals 4 days after a myocardial infarction, they found that 4.4% of respondents had exercised intensively within the hour before the infarction. Patients who were physically active less than once per week had a 107-fold greater risk of myocardial infarction during periods of intense exercise than during periods of rest (relative risk [RR] = 107, 95% confidence interval [CI] = 67–171); those who were physically active at least five times per week had only a 2.4-fold greater risk of myocardial infarction during periods of intense exercise than during periods of rest (RR = 2.4, 95% CI = 1.5–3.7). More recent studies have also shown a positive correlation between more intense physical activity and the increased occurrence of stroke, acute myocardial infarction, and sudden cardiac death [34–36]. Regardless of age, intense physical activity may trigger myocardial infarctions, especially in those who are unaccustomed to exercise [37, 38].

#### Recommendation 3

**Table:** 

A PPE should be offered at 1–5-year intervals, depending on the individual’s risk profile, performance level, sport type, and intensity	
*Recommendation grade:* moderate	⇑
*Strength of consensus:* 100% (strong)	
*Justification:* The available data are insufficient to determine specific time intervals between examinations. The consensus group assumes that such a recommendation depends on individual risk factors, such as age and fitness level (especially cardiorespiratory fitness level). Furthermore, it concluded that annual screening is not sensible, feasible, or deserving of prioritization	

An initial examination is the best way to obtain a medical overview of an individual. Follow-up examinations are based on personal preference and, primarily, individual risk. For example, the rate of fatal or serious incidents and the risk of injury during sports increases mainly with intensity [3–5]. Few (if any) experts would argue that Nordic walking should not be judged differently from alpine skiing. Therefore, a PPE is not logical in every case. In addition, follow-ups to check the success of the training recommendations or to make adjustments are desirable but not part of the overall PPE.

#### Recommendation 4

**Table:** 

Use the PPE results to derive individualized exercise and training recommendations	
*Recommendation grade*: strong	⇑⇑
*Strength of consensus:* 100% (strong)	
*Justification:* The Guideline Development Group agrees that a strong recommendation is appropriate here, especially as PPE results can be used for individual counseling	

Despite the known health benefits, more than 40% of adults in Germany are physically inactive or insufficiently active (i.e., they engage in < 150 min of moderate-intensity or < 75 min of high-intensity activity per week). Sedentary behavior is defined as less than < 5000 steps per day [39]. General practitioners may play a significant role in health counseling, patient motivation, and advising patients on how to change their lifestyles [40, 41]. Offering individualized exercise and training recommendations in a preventive medical check-up (PPE) could also contribute to greater use of this service. Information from sports history can be used as a basis for the recommendations (see Recommendation 9). Such an exercise prescription should consider frequency, intensity, time, and type (the FITT principle; [14]). Volume and progression (the FITT-VP principle) can also be considered, where the duration and intensity of the training gradually increase within the framework of a progressive transitional phase.

#### Recommendation 5

**Table:** 

Techniques to support behavioral change should be used when communicating exercise recommendations	
*Recommendation grade:* moderate	⇑
*Strength of consensus:* 100% (strong)	
*Justification:* Increasing evidence shows that communication techniques to support behavioral change are important in the medical field. However, no gold standards are currently available as the techniques and applications examined in literature are heterogeneous. Therefore, medical practitioners could use PPEs to motivate individuals to adopt active lifestyles by adopting behavioral change techniques. Although this may appear to contradict the primary focus of this guideline, the Guideline Development Group decided to include this recommendation	

Although in Germany the promotion of an active lifestyle is part of health check-ups, this low-threshold approach is rarely used in practice (about 20%) [42]. Therefore, offering a PPE may motivate an active lifestyle if it is supported by behavioral change techniques [43–45]. A recent Finnish study achieved increased physical activity among patients with diabetes through appropriate counseling in primary healthcare settings [46]. Qualified counseling support appears to be particularly crucial for increasing physical activity among less-active patients [47]. Various techniques exist for both phases, including goal setting, action planning (concrete “what, when, where, with whom” plans), barrier management (“If obstacle X occurs, I react with strategy Y to achieve my goal”), and self-observation (e.g., keeping an action diary or adjusting the action plan if the goals are not achieved), all of which have proven effective (c.f. [48]). Motivational interviewing (MI) is particularly emphasized in literature as a potential communication method for promoting an active lifestyle [40]. MI generally prioritizes patients’ autonomy, desires, goals, and visions. An MI intervention comprises four phases: (1) building a relationship (engaging), (2) goal setting (focusing), (3) strengthening motivation (evoking), and (4) planning the implementation (planning). MI is based on principles such as empathy, identifying discrepancies, managing resistance, and strengthening confidence in change. Recent studies indicate that MI can result in health improvements even under the time constraints that are often encountered in primary care settings [49].

#### Recommendation 6

**Table:** 

A PPE should be conducted by a specialized physician with an additional qualification in sports medicine	
*Recommendation grade*: moderate	⇑
*Strength of consensus:* 80% (moderate)	
*Justification:* The available data are insufficient to specify a specific qualification as a prerequisite for conducting a PPE. However, owing to the interdisciplinary nature of the corresponding examination, the Guideline Development Group assumes that in addition to the specialist knowledge associated with a “sports medicine” qualification, it guarantees a higher level of quality and expertise in conducting such screenings. This notion applies especially to cases where individuals plan to increase their physical activity levels, as knowledge of sports medicine appears necessary to properly assess individuals’ levels of physical exertion and resilience	

Currently, medical specialists in sports medicine exist in only a few countries. The new EU regulations for sports and exercise medicine (Article 26 of Directive 2005/36/EC [16]) underscore the need for evidence-based recommendations for PPEs. In Germany, for a formal qualification in sports medicine, a curriculum of 240 h of theoretical knowledge and 120 h of practical experience in sports medical care of a club is required. In our survey, several potential participants considered this additional qualification relevant for the physician because it guarantees the quality and effectiveness of a preventive medical check-up intended to detect at-risk individuals. Potential participants also stressed the importance of receiving individual training recommendations, and several survey respondents considered these more trustworthy when received from a qualified sports physician. Others found easy access to a PPE more important than the provider’s formal qualifications. Specific sports medicine questions, especially in performance-oriented areas, require specialized knowledge owing to the cross-sectional nature of the sports medicine field.

### Algorithm for the PPE

**Fig. 3:**
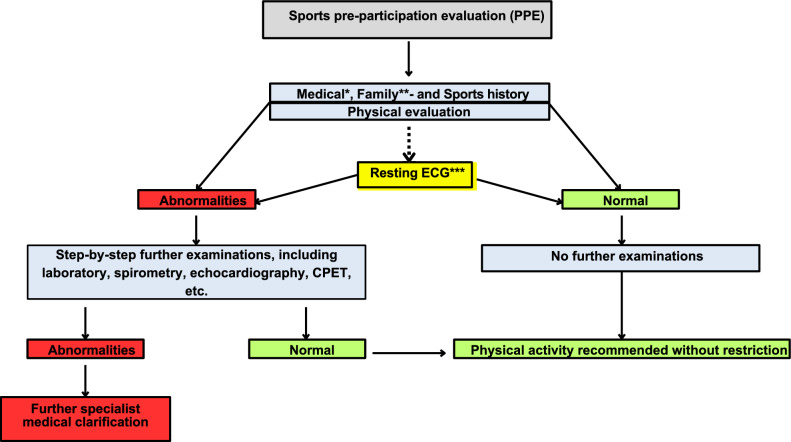
The new consensus-based algorithm for the PPE for healthy adults. *Including symptoms, presence of diseases, and risk factors (e.g., a Systematic Coronary Risk Evaluation 2 [SCORE2] > 10%); **including the sudden cardiac death of close relatives aged < 60 years; ***if no recent resting ECG is available or if the medical history and/or physical evaluations indicate a need for one. *ECG* electrocardiogram, *CPET* cardiopulmonary exercise testing

The new consensus-based algorithm procedure for the PPE is shown in Fig. 3. The results of individuals’ medical, family, and sports histories and physical evaluations by a qualified physician allow them to be divided into individuals with abnormal and normal findings. An evaluation is considered abnormal when the findings indicate the possibility of risks in terms of high-intensity exercise (e.g., a high-risk profile on the Systematic Coronary Risk Evaluation 2 [SCORE2; > 10%]), alarming symptoms (e.g., pain and syncope), or abnormal findings upon physical examination. In such cases, further examinations should be conducted to clarify the findings. A resting electrocardiogram (ECG) is recommended if no recent ECG is available or if the individual’s medical history and/or physical evaluations indicate that one is needed. An evaluation is considered normal in the absence of the abovementioned findings. The new process diverges from the previously available algorithms of the EFSMA [7] and ACSM [10] in that it does not focus on athletes or exclusively cardiovascular diseases.

### Medical History and Physical Examination

#### Recommendation 7

**Table:** 

A standardized medical history form should be used in the PPE. It should include: • Personal and family medical history • Sports history • Individual risk-factor profile • Medication history • Nutritional history • Gynecological history • Vaccination status • Participation and results of previous check-ups • Previous injuries and/or surgeries	
*Recommendation grade*: moderate	⇑
*Strength of consensus:* 100%	
*Justification:* The (predominantly indirect) evidence and the benefit–harm–cost balance supports a moderate recommendation for a standardized medical history form	

The medical history should include relevant information about the individual’s previous sporting experience, as well as physical activity, nutrition status, health, dietary behavior, well-being, and vaccination status. This comprehensiveness seeks to identify any risks to the individual (latent) or their family that could arise through intensive sports, based on the 11th edition of the ACSM’s guidelines [10]. It includes recommendations on various questions for individuals interested in amateur and recreational sports, covering previous diagnoses, interventions, physical and laboratory examination results, symptoms, illnesses, hospitalizations, (overuse) injuries or other orthopedic diagnoses, medication, stimulants, and sporting and occupational activity, completed by a family history [10]. Overall, individual risk should be assessed on the basis of validated scores (see Recommendation 8). The US-based National Athletic Trainers’ Association also emphasizes recording individuals’ intake of medication and dietary supplements [50].

This information is used to determine the need for follow-up examinations (e.g., resting ECG, echocardiography, and stress tests). Since 2022, the American Medical Society for Sports Medicine has also recommended asking about coronavirus disease-2019 (COVID-19) history to document previous severe acute respiratory syndrome-coronavirus-2 (SARS-CoV-2) infections and any past or current symptoms, diagnoses, sequelae, and persistent or new symptoms [51, 52].

The EFSMA recommendations regarding medical histories for elite athletes are comparable [53]; EFSMA also offers a corresponding examination form for recreational sports (Supplementary Material 3).

#### Recommendation 8

**Table:** 

Use a validated score (e.g., Arriba and SCORE2) to assess cardiovascular risk from the age of 35 years	
*Recommendation grade*: strong	⇑⇑
*Strength of consensus*: 100% (strong)	
*Justification:* The evidence and benefit–harm balance support a strong recommendation. The use of a validated instrument also objectifies the risk assessment; however, the available data are insufficient to recommend one gold standard among the validated scores	

The ESC recommends evaluating cardiovascular risk (e.g., using the SCORE2 [54] or SCORE2 for Older Persons [SCORE2-OP] [55, 56]) before engaging in high-intensity physical activity, especially for those aged ≥ 35 years [9]. No further cardiovascular examination is recommended for those with low or moderate risk who lack family risk factors [10, 54]. According to the ESC, further diagnostic procedures (e.g., cardiopulmonary exercise testing [CPET]) can be considered for those at high risk (e.g., SCORE2 > 10%) with a high level of planned exercise intensity [54].

The German national care guidelines for coronary heart disease (CHD) recommend specific diagnostics for the general population once the risk of current CHD reaches 15% [57]. The risk of current CHD is not the same as the risk of suffering a myocardial infarction or stroke within the next 10 years—it is relatively greater.

SCORE2 combines the morbidity–mortality risk of myocardial infarction, apoplexy, and cardiovascular death to assess risk from a primary prevention perspective [58]. In addition to sex and age, the SCORE2 calculation includes total cholesterol, high-density lipoprotein, systolic blood pressure, and smoking. A corresponding instrument was developed for individuals aged > 70 years called SCORE2-OP [55, 56]. In general practice, the Arriba score, which is comparable to the SCORE2-Germany or Prospective Cardiovascular Münster (PROCAM) score in terms of informativeness, is predominantly used [59]. Its algorithm is based on the Framingham data and considers the presence of antihypertensive therapy, diabetes mellitus, and hemoglobin A1c (HbA1c), as well as corresponding events in individuals’ or their family histories and the factors mentioned in the SCORE2. This algorithm predicts the risk of a heart attack or stroke within the subsequent 10 years. Additional scoring mechanisms can be found online [60].

#### Recommendation 9

**Table:** 

A sports history should include questions about the following factors: • Frequency, intensity, time, and type (FITT) • Previous sporting experience • Goals of the physical activity • Environmental conditions during the sport (e.g., heat, cold, or altitude) • Complaints at rest and during exercise • Individual sport-related risk profile • Aids (e.g., visual aids) or mobility restrictions	
*Recommendation grade*: moderate	⇑
*Strength of consensus:* 100% (strong)	
*Justification:* There is no robust evidence in literature on the specific structure of a patient’s “sports history”. However, the Guideline Development Group assumes these aspects are relevant in the context of risk assessment and counseling	

Knowing how much physical activity an individual performs (if they are currently active) is essential to assess their risk and offer tailored advice [27, 61]. The individual’s objectives, previous experiences, complaints, sport-related risks (e.g., arterial hypertension and visual impairments), any aids required, and mobility restrictions should be recorded. As is already done in organized sports and for athletes, questions about heat acclimatization are recommended as part of the medical history [62], including risk factors, fluid intake, training intensity, and previous reactions to extreme environmental conditions [62, 63]. In exercise counseling, the FITT principle should also include volume (total amount of exercise per intervention) and progression (changes in exercise program difficulty over time, e.g., an intervention that begins at moderate intensity and progresses to high intensity over several weeks) [10].

**Table 2:** Recommended components of the physical examination

Examination	Exemplary entities
Collect anthropometric data (measure height, body weight, and waist circumference; calculate body mass index, body mass changes, and waist-to-height ratio) and record body fat percentage and fat-free mass	Assess body mass status (e.g., indications of RED-S), obesity, body mass changes, and body composition (e.g., sarcopenia and fat distribution pattern)
Measure heart rate and blood pressure (at least once on both sides)	Arrhythmia, arterial hypertension, and aortic isthmus stenosis
Examine the heart and lungs (sitting, standing, and lying)	Vitium cordis (e.g., patent foramen ovale or mitral valve prolapse), COPD, and pulmonary fibrosis
Assess vascular status	PAD and evidence of aneurysm
Examine the abdomen	Fatty liver, hernias, and portal hypertension
Examine the musculoskeletal system, including the range of motion and stability of the large joints, spinal mobility and pain on motion and palpation, and muscle status (including flexibility, function, and tone)	Increased risk of injury (e.g., joint instability) and reduced risk of overload
Examine the nervous system (e.g., gait pattern, reflex status, sensitivity, and cranial nerve status if necessary)	Tendency to fall, impaired coordination, and protective reflexes
Examine the sensory organs, including a visual acuity test with eye charts	Impaired hearing and/or vision
Inspect the skin and mucous membranes	Infectious diseases, anemia, eczema, and malignancies
Inspect the oral cavity and record dental status	Status quo, chronic gingivitis, and bulimia nervosa
Assess lymph node status	Acute infection and systemic disease

*RED-S* relative energy deficit in sport, *COPD* chronic obstructive pulmonary disease, *PAD* peripheral artery disease

#### Recommendation 10

**Table:** 

Determine whole-body status as part of the PPE, which should be based on the recommendations in Table 2	
*Recommendation grade*: strong	⇑⇑
*Strength of consensus*: 100% (strong)	
*Justification:* The evidence and benefit–harm balance support a strong recommendation, although the individual examination components are supported to varying degrees	

For recreational sports, the ACSM recommends a physical examination consisting of anthropometry, pulse and blood pressure measurement, auscultation of the lungs and heart, palpation of the foot pulses and the abdominal and femoral arteries, palpation of the abdomen, and a visual inspection for the presence of tendon xanthomas or skin xanthelasma; a follow-up examination based on anamnestic complaints; and a basic neurological examination [10]. For athletes, an examination of visual acuity, the ears, nose and throat area, skin, body composition, and the musculoskeletal system is also recommended [7, 30, 50]. A dental examination is only recommended by the EFSMA guidelines [64]. The examination components (Table 2) were discussed during the consensus-building process. The selection of specific examinations and their scope should be guided by information from individuals’ medical or sports history.

#### Recommendation 11

**Table:** 

Patients with joint swelling, joint pain, relevant range-of-motion restrictions or large joint instability, as well as movement or knocking pain in the spine (with or without neurological deficits), should be referred to a specialist for further evaluation (preferably a specialist in orthopedics/trauma surgery or physical and rehabilitative medicine)	
*Recommendation grade:* moderate	⇑
S*trength of consensus*: 100% (strong)	
*Justification:* There is insufficient evidence for a strong recommendation. However, the benefits appear to outweigh the risks and effort involved. Furthermore, an orthopedically experienced sports physician who feels confident in performing an orthopedic examination and clarification should not be denied this opportunity	

While the internal medicine portion of the PPE aims to prevent fatal incidents, fatal injuries that are attributable to underlying orthopedic conditions are extremely rare. In contrast, abnormal musculoskeletal findings are relatively common during PPEs and represent the main reason for sports restrictions [65]. Therefore, orthopedic screenings serve primarily to minimize the risk of exacerbating previous conditions and prevent new acute or overuse injuries. Numerous epidemiological studies have shown that both the former (especially in team sports) and latter (especially in endurance sports) injuries are very common in popular recreational sports. In the SAFER (Strategies to reduce Adverse medical events For the ExerciseR) XIII study involving 21,824 recreational cyclists, Du Toit et al. found that 2.5% of participants suffered an overuse injury yearly [66]. In competitive running, rates as high as 79.3% have been reported, with significant adverse effects on training workload and health [67, 68]. These data provide a good basis for counseling patients regarding the prevention of overuse injuries.

The examination must primarily detect and consider tendon, bone, and joint disorders, as well as muscular imbalances, which certain sporting activities can exacerbate. If possible, the physical examination should be conducted at least 6 weeks before physical activity to accommodate further examinations, treatment, or rehabilitation measures if warranted [69]. This recommendation is intended to assist physicians in detecting relevant findings; it should explicitly not be considered a barrier to sports. In the stakeholder survey of sports physicians, only 67% expressed feeling sufficiently qualified to perform the orthopedic component of a PPE. However, the importance of the examination and its performance by a trained examiner is emphasized in literature [18]; therefore, a further evaluation of any abnormal findings would also be important.

During the physical examination, the factors “joint swelling,” “pain on movement or palpation,” and “joint instability” are important indicators of relevant articular or osseous diseases, which should be considered when choosing suitable exercise types [69]. “Pain” is not to be understood as diffuse pain on palpation or pain from muscle tension but instead as relevant joint pain or localized knocking pain in the spine. An orientating neurological examination completes the spine and limb evaluation. As with the abovementioned factors, lateral differences in strength of more than 10–20% require further evaluation [70].

Other tests for which sufficient data exist to allow the detection of an increased risk of injury in athletes—such as the dynamic knee valgus in the single-leg squat test for a previous anterior cruciate ligament injury [71] and the tibial edema test for the early detection of medial tibial stress syndrome [72]—exceed the scope of a general preventive examination in the general population and, therefore, should be reserved for athletes or conducted only as part of further evaluation (by specialists if necessary) in cases of relevant abnormalities.

#### Recommendation 12

**Table:** 

Individuals with arthroplasties or serious injuries in their medical history (e.g., vertebral fractures or severe/recurrent joint injuries) should be regularly monitored by a specialist, preferably in orthopedics/trauma surgery or physical and rehabilitative medicine	
*Recommendation grade*: moderate	⇑
*Strength of consensus:* 91% (moderate)	
*Justification:* There is insufficient evidence for a strong recommendation. Presumably, most individuals benefit from this recommendation because the advice regarding their physical activity will be based on a thorough examination and important information from their medical history	

Numerous studies have shown that previous injuries are among the most important risk factors for new injuries [73–76]. Therefore, it is important to consider these in a preventive examination to limit recurrences or consequential harm. However, the Guideline Development Group considers in-depth assessments beyond the scope of a preventive examination; for example, the fit and loading capacity of an endoprosthesis or the status of a previously highly damaged joint often require additional imaging and documents (e.g., X-rays, surgical reports, and an arthroplasty passport). It is useful to consult a specialist for competent advice on exercise dosages and recommended types of sports in such cases. Modern artificial joints can withstand high physical loads and their survival rates are even higher in active individuals (c.f. [77]). It is currently assumed that almost any sporting activity can be performed with a firmly anchored, well-integrated endoprosthesis. It is the attending specialist’s responsibility to confirm this, although there are no clear recommendations regarding the intervals at which this specialist supervision should occur.

### Further Examinations

Owing to a lack of evidence in the context of further examinations for recreational and popular sports, the following recommendations (and their respective strengths) are based primarily on the guidelines and consensus documents from competitive sports and underlying primary literature. Even for these populations, the recommendations from various professional societies are inconsistent (see [9]) in terms of, for example, resting ECG, echocardiography, and stress tests. Therefore, in even larger and more heterogeneous general populations, feasibility, cost–benefit balance, examiners’ qualifications, and avoiding overdiagnosis must be particularly considered. Potential additional examinations are based on an individual’s medical history and physical examinations and depend on the availability and timeliness of previous findings (e.g., laboratory tests and resting ECG).

#### Laboratory Tests

##### Recommendation 13

**Table:** 

If necessary, a blood count, plasma glucose, HbA1c and lipid status, liver/kidney values, electrolyte concentration, and urine status can be determined as part of a PPE	
*Recommendation grade*: weak	⇔
*Strength of consensus:* 100% (strong)	
*Justification:* The available data are insufficient to recommend specific laboratory tests. In principle, however, the Guideline Development Group assumes that the mentioned parameters are helpful if there are corresponding indications or risk constellations. In addition, lipid status is required to determine some scores. However, this is often available from other examinations, particularly the general health examination	

Selected laboratory examinations are logical for some groups, such as athletes (e.g., iron deficiency in female athletes, vitamin D, or relative energy deficit in sport [RED-S]), or regions, such as southern Germany (thyroid-stimulating hormone [TSH]), but not generally necessary for a PPE. These should only be recommended on the basis of abnormalities observed in an individual’s history or physical examination [9]. The examples in Recommendation 13 are based on general screening tests, Germany’s national type 2 diabetes care guidelines [78], and the scores recommended in Recommendation 6. Nonetheless, even in organized sports, the routine screening of urine samples or blood counts is not recommended in the USA; it is recommended only if possible deficiencies are indicated (e.g., hemoglobin and ferritin in the case of a history of anemia or elevated cholesterol or lipid levels) [50].

#### Cardiovascular Examinations

In the context of a PPE, cardiovascular examinations are intended to detect abnormalities, which should then be assessed by a specialist (e.g., a sports cardiologist).

##### Recommendation 14

**Table:** 

A 12-lead resting ECG should be performed as part of each PPE	
*Recommendation grade*: moderate	⇑
*Strength of consensus*: 100% (strong)	
*Justification:* The limited evidence, cost–benefit analysis, and considerations of likely consequences (e.g., follow-up examinations after false-positive findings) support a moderate recommendation. Therefore, a resting ECG should be performed if no recent resting ECG is available or the medical history and/or physical examination indicate one	

The demand to integrate a resting ECG into the PPE of competitive athletes, based mainly on decades of Italian Working Groups’ experience, can be attributed primarily to the increased presence of arrhythmogenic right ventricular dysplasia [79]. Harmon et al. demonstrated that an ECG has a significantly greater sensitivity to predict cardiac disorders (94%) than a medical history (20%) or physical examination (9%) [80], making it a particularly valuable screening tool for identifying abnormalities that could increase the risk of sudden cardiac events. The rate of false positives is also lower with an ECG (6%) than a medical history (8%) or physical examination (10%), reducing the use of unnecessary follow-up tests.

Integrating a resting ECG into athletes’ PPEs reduced the annual incidence of sudden cardiac death in Veneto, Italy, by 89% [81]. In addition, special attention should be paid to other arrhythmogenic conditions, such as Brugada syndrome, epsilon potential in arrhythmogenic right ventricular dysplasia, or long QT syndrome due to congenital or acquired causes (e.g., psychiatric medications, such as amitriptyline or citalopram, or antibiotics, such as macrolides). However, this type of diagnosis usually requires more in-depth knowledge (e.g., a sports cardiologist) [82].

The extent to which this applies to the general population, as well as to athletes from other countries, has yet to be conclusively determined. It is undoubtedly justified only if no current resting ECG (e.g., within the last 12 months) exists or if the medical history and examination findings warrant performing one. A resting ECG combined with a comprehensive medical and family history offers advantages in that the associated costs and effort are low while the potential information gain is high [83].

While a sensitivity and specificity of > 90% for abnormal findings on resting ECGs suggest its clinical dependability, the ratio of true- to false-positive abnormal findings for relevant heart diseases is typically 1: > 400 (positive predictive value) owing to these diseases’ low incidence [84]. Therefore, for every identified case of disease, several hundred ultimately harmless ECG findings and corresponding complex follow-up examinations can be expected.

The practical implementation and knowledge required to adequately classify potential sports-related findings must also be considered because, unlike in the general population, changes in resting ECG are often identified in athletes (c.f. [85, 86]). This knowledge appears essential for reducing false-positive findings of relevant cardiac pathologies in resting ECGs. Conceivably, doctors without sports cardiology training could use digital or artificial intelligence-based tools to interpret athletes’ ECGs. However, it is currently impossible to say whether this would also apply to the general population.

##### Recommendation 15

**Table:** 

Perform an echocardiogram as part of a PPE if there is reason to suspect structural heart disease	
*Recommendation grade*: strong	⇑⇑
*Strength of consensus:* 90% (moderate)	
*Justification:* The Guideline Development Group believes the cost–benefit analysis supports a moderate recommendation	

Echocardiography is not recommended as a routine screening tool [83]. However, the ACSM [10] recommends it for diagnosis when corresponding symptoms or known heart disease exist. Some structural cardiac abnormalities that are difficult to detect during a physical examination or resting ECG can often be recognized with additional tests. For example, around 10% of sudden cardiac deaths in young athletes can be attributed to structural heart diseases without conduction disturbances [87]. For example, Donati et al. compared a standard screening (medical history, physical examination, ECG, and stress testing) with an advanced screening (including echocardiography) [88]. They showed that 9.1% of patients who exhibited no abnormalities in the standard screening had cardiovascular issues, such as patent foramen ovale and mitral valve prolapse, that echocardiography revealed. This finding highlights the added value of echocardiograms in identifying otherwise undetected conditions. Therefore, transthoracic echocardiography (TTE) is supported in such populations. However, it has not yet been established owing to a lack of practicality, a lack of time, and an unfavorable cost–benefit ratio, among other reasons. Halasz et al. [87] demonstrated that a structured procedure was cost-effective in elite athletes, given the costs of sudden cardiac death, when assuming an examination duration of only 10 min [87]. However, this finding is hardly transferable to recreational and popular sports because the TTE tends to take longer, even for “normal” examinations. Furthermore, it fails to overcome the problem of limited knowledge and resources for performing the examination. Consequently, echocardiography should not be routine in PPEs and should only be performed if there is reasonable suspicion of possible structural heart disease.

##### Recommendation 16

**Table:** 

An exercise ECG should be performed as part of a PPE if warranted by the PPE findings, the individual risk profile, possible exercise-induced symptoms, the type of sport in question, and the performance level and intensity	
*Recommendation grade:* moderate	⇑
*Strength of consensus:* 100% (strong)	
*Justification:* The Guideline Development Group finds that the cost–benefit analysis and the limited evidence in the area of sports medicine screening support a moderate recommendation. However, the individual performance/fitness assessment is emphasized (see also Recommendation 17)	

Exercise tests have been used for decades to provoke and identify myocardial ischemia. They are also used to detect cardiovascular disease (especially CHD) and assess physical performance, exercise tolerance, exercise-related symptoms, chronotropic competence, possible arrhythmias, and responses to medical interventions [89]. However, exercise ECGs are viewed critically for the diagnosis of hemodynamically relevant coronary stenoses owing to their low sensitivity (58%) and specificity (62%) [90–92]. In sensitivity and specificity calculations, most of the included individuals had at least suspected coronary artery disease (CAD). Therefore, the applicability of these metrics to a seemingly healthy population remains unclear. Furthermore, the quality of the test depends on whether maximal cardio-respiratory stress was achieved, among other factors, such as pre-test risk or the presence of stable CAD. Ermolao et al. demonstrated that the systematic use of a maximal exercise test as part of a screening protocol was more sensitive in detecting cardiovascular diseases [12]. Microvascular dysfunction may also be present even when no hemodynamically relevant coronary stenoses are detected [93]. Therefore, the aim should be to achieve maximum exercise capacity during the implementation, even among older adults.

Performing a stress test is not recommended uniformly in PPEs. According to the ACSM guidelines [10], most (older) adults do not require an ergometric stress test before engaging in moderate physical activity. There are also few reliable studies (especially prospective studies) in literature showing the benefits of such a test as part of a PPE conducted for reasons other than planned high-intensity exercise and the assessment or determination of performance [94]. However, the EFSMA suggests a complete clinical assessment, including ergometric stress tests, for older adults and physically inactive individuals who wish to participate in intense training [94]. The German pocket guideline [95] also recommends an exercise ECG for some patients to assess their exercise tolerance, symptoms, arrhythmias, blood pressure behavior, and risk for cardiovascular events. Therefore, an exercise ECG that considers examination findings, individual risk profiles, possible exercise-induced symptoms, sport type, performance level and intensity, and pre-test probability should be used.

Even if these aspects essentially indicate the possible occurrence of CHD in individuals with (predominantly stress-dependent) chest pain, a corresponding pre-test risk is practically useful. The Marburg Heart Score for chest pain is also used in primary care, which combines clinical symptoms and physical evaluation findings in cases of acute breast pain. Thresholds vary for secondary care (c.f. National Disease Management Guideline for Chronic CHD [57]).

#### Determination of Physical Fitness

##### Recommendation 17

**Table:** 

A CPET can be used to determine cardiorespiratory fitness and provide training recommendations/guidance as part of a PPE	
*Recommendation grade:* weak	⇔
*Strength of consensus*: 100% (strong)	
*Justification:* The Guideline Development Group agrees that the evidence is insufficient to recommend a CPET as standard. Nonetheless, such a test can provide helpful findings, especially for tailoring and managing training recommendations. Therefore, it is regarded as an optional component of a PPE	

In addition to its diagnostic usefulness and excluding possible exercise-induced abnormalities, a CPET can determine individual cardiorespiratory performance and fitness. This determination supports personalized training advice to facilitate safe and effective exercise design [96]. Fitness is one of the most critical prognostic markers for reduced mortality and morbidity associated with all noncommunicable diseases (e.g., [97]), as well as a vital sign alongside heart rate, blood pressure, body temperature, and respiratory rate [98]. An observational study of 22,878 asymptomatic participants (mean age: 47 years, 28% female) who were followed up for about 9 years found that the relative mortality risk was nearly 36-fold greater in those with a high European System for Cardiac Operative Risk Evaluation (EuroSCORE; ≥ 5) and low cardiorespiratory fitness (defined as a maximum endurance capacity of < 11 METs) than in those with a low EuroSCORE (< 5) and high cardiorespiratory fitness (≥ 11 METs) [99]. However, if the at-risk individuals were “fit” with the same EuroSCORE, the RR was only 8.5. In addition, a CPET with breath gas measurements is suitable for the differential diagnosis of exercise-induced dyspnea because it helps distinguish between pulmonary-obstructive and pulmonary-vascular and cardiac limitations [100]. In Germany, power output during bicycle ergometry is typically measured in watts. While watts measure the rate of energy output, converting them into METs helps to clarify the actual energy expenditure during an activity (METs = watts/[body weight (kg) × 3.5]).

##### Recommendation 18

**Table:** 

Muscle strength can be determined to measure muscular fitness (e.g., hand grip strength) as part of a PPE	
*Recommendation grade:* weak	⇔
*Strength of consensus*: 100% (strong)	
*Justification:* The available data are insufficient to make a strong recommendation. In principle, however, the Guideline Development Group assumes that muscular fitness will play an increasingly important role over time as a general surrogate parameter and for specific training recommendations	

The health benefits of improving muscular fitness are now well-documented ([1]; summarized in [18]). Muscular fitness includes strength, speed, isometric strength, and endurance [10, 101]. Greater muscular strength is associated with a significantly better cardiometabolic risk profile, reduced all-cause mortality, fewer cardiovascular events, a lower risk of developing physical functional limitations, and a lower risk of nonfatal diseases [10, 101]. Therefore, muscular fitness can be measured in a PPE not only as a reflection of the patient’s condition but also as a basis for appropriate training recommendations.

In literature, muscular fitness is usually determined by measuring hand grip strength. Dodds et al. [102] developed percentiles for children, adolescents, men, and women aged 4–90 years on the basis of almost 50,000 participants from 12 population studies in Great Britain. However, muscle-specific tests are required to develop tailored training recommendations, such as the maximum weight that can be moved for a defined number of repetitions (e.g., 1-, 3-, 5-, or 10-repetition maximums) [102]. Other tests (e.g., the “timed up and go” or “chair-rise” tests) can also be used in this context, although they are used primarily for older individuals [103].

### Additional Aspects

#### Recommendation 19

**Table:** 

Perform laboratory and instrumental examinations as part of a PPE that go beyond Recommendations 13–18 only in justified individual cases	
*Recommendation grade:* strong	⇑⇑
*Strength of consensus*: 100% (strong)	
*Justification:* The Guideline Development Group considers the evidence for investigative procedures other than those listed in Recommendations 13–18 insufficient to recommend them as standard for PPEs. In its opinion, besides the limited evidence, the benefit–harm–cost balance strongly supports performing them only in justified individual cases	

Further investigations, such as laboratory tests (i.e., iron, ferritin, or vitamin D), echocardiography, or lung function, should be based on possible findings from individuals’ medical history or preceding diagnostics. However, literature offers no evidence regarding the potential benefit of performing additional diagnostic procedures as part of a PPE. When corresponding symptoms are present, such as fatigue, reduced performance, or specific anamnestic indications, the search for a possible cause of illness or previously undiagnosed health disorders should be prioritized over fitness for sports. Providers should also remember that a low pre-test risk increases the proportion of false-positive findings with an additional, avoidable need for clarification [104].

#### Recommendation 20

**Table:** 

During the PPE and counseling, assess each individual’s risk of danger to themselves and others and consider the possible worsening of previous injuries	
*Recommendation grade:* strong	⇑⇑
*Strength of consensus*: 100% (strong)	
*Justification:* Given its objectives, assessment and counseling about the risk of danger to oneself and others is an essential part of the PPE. Therefore, the Guideline Development Group believes that the benefit–harm–cost balance supports a strong recommendation	

Assessing possible risks to the health of the individual or others is a central element of a PPE. While the recommendations are focused on athletes (c.f. [9]), the Guideline Development Group discussed including this recommendation to, again, explicitly emphasize the individual’s status quo.

The American Academy of Pediatrics provides a list of guiding questions that offer a solid foundation for such assessment and can function as a checklist (Box 7 in [30]):

- Does participation put the athlete at risk of illness or injury above the inherent hazards of the activity?
- Does participation increase the risk of injury or illness to other participants?
- Will treatment of the underlying condition allow safe participation (medication, rehabilitation, bracing, or padding)?
- Can limited participation be allowed while treatment or evaluation is completed?
- If medical eligibility is denied for certain sports because of medical or safety concerns, can the athlete safely participate in other activities or sports?

## Discussion

The benefits of regular physical activity far outweigh its risks [3, 105, 106]. However, although fatal events such as sudden cardiac death or acute myocardial infarction are very rare, participation in exercise is associated with an increased short-term risk of musculoskeletal injuries and cardiovascular complications [33, 105]. This observation highlights the importance of a PPE designed to identify at-risk individuals to help prevent fatal cardiac or musculoskeletal events during or after exercise. While the existing guidelines mainly focus on examining (elite) athletes, this consensus-based guideline is designed for assessing healthy adults who plan to start or return to intensive sports or exercise. Although no sufficiently robust or specific evidence currently exists for the positive effects of such examinations on patient-relevant outcomes or their optimal sensitivity/specificity [107, 108], this guideline seeks to balance expert knowledge and feasibility in medical practice with the available evidence.

There was broad consensus on the importance of taking detailed personal, family, and sports histories and conducting a thorough physical evaluation. Depending on the results, further procedures could be implemented as necessary. Unlike previous guidelines that primarily focused on PPEs in athletes, this guideline does not mandate the performance of a resting ECG. Nonetheless, decades of experience among Italian working groups have shown that including a resting ECG in PPEs for athletes has contributed to reducing the number of sudden cardiac deaths in that population [73, 100]. While these results are promising, the generalization of this approach to other countries and the general population is controversial, and caution is needed when applying these findings beyond their original context.

The challenge of implementing PPEs for a broader population lies in the need for knowledge and competencies across various medical disciplines. For example, not only the musculoskeletal system but also the individual’s cardiovascular status must be examined. While the EU has included a specialization in sports medicine in its directives for practitioners, this qualification is not universally accessible. In Germany, sports medicine knowledge is primarily taught through a course-based system and, to a lesser extent, in sports medicine institutes. Despite these options, there is no comprehensive, nationwide coverage. To what extent technical advancements (e.g., the use of artificial intelligence in ECG analysis) might support more accurate assessments of sports-related changes in individuals remains an open question. However, these advancements offer promising potential to close the existing gaps in practitioner knowledge [109, 110].

In addition, the contents of PPEs can and should ground exercise-related counseling (e.g., FITT-VP) to promote physical activity meaningfully. Physical performance is important in assessing health status [18, 19]. Alongside general exercise-related counseling, preventive advice on avoiding stress-related damage and injuries can be given. This approach explicitly aims not to create additional barriers to sports participation but rather to enable safe and inclusive access.

### Strengths and Limitations

One key strength of this guideline was basing its recommendations on a synopsis of the available guidelines and consensus papers from numerous professional societies and associations. Discussing these diverse perspectives contributed significantly to the development of robust recommendations, as demonstrated by the interdisciplinary composition of the 16 specialist societies in family medicine, general and specialized internal medicine (endocrinology, cardiology, angiology, and pulmonology), orthopedics, and rehabilitation, as well as associations (including sports organizations and disabled sports associations) and experts in other relevant fields (ophthalmology, nutritional sciences, and neurology/psychiatry). Surveying medical professionals and potential participants/patients offered a user perspective and enriched the discussions. That said, whether the recommendations or their strength would have varied if the survey participants had been allowed to vote in the guideline creation process remains speculative.

One main limitation of this guideline was the paucity of available data. The synopses mainly described athlete-based recommendations—mostly at the elite level—without randomized controlled trials [9]. Furthermore, no intervention study has supported screening for injury risk [111]. Presently, no cost–benefit analysis of this PPE or the number needed to prevent (NNP) are known. Any such evidence is available only in the context of competitive sports. Therefore, registries and population-based studies are urgently needed to demonstrate the benefits of PPEs for identifying at-risk individuals and promoting and counseling physical activity among the general population. Their initiation should be encouraged sooner rather than later so that, if necessary, the PPE algorithm can be adapted to incorporate their findings.

Another potential limitation is that the resources for conducting PPEs may be limited in less-developed countries. In addition to structural and financial resource constraints, the different qualifications available in different countries will influence the implementation of such examinations. While the Guideline Development Group agrees that further training in sports medicine is desirable to ensure high-quality examinations, it is not available everywhere. Therefore, regional differences must be considered when adapting and implementing the guidelines in other countries. In other words, our recommendations describe PPE requirements solely from a medical perspective; the extent to which healthcare providers can meet and finance these needs promptly remains to be determined.

### Recommendations for Future Research

Future studies must assess the extent to which this guideline can reduce or prevent fatal events, and its design, outcomes, and practical feasibility, as well as the target populations reached, should be analyzed regularly. Whether a PPE for individuals participating in recreational/health sports provides benefits in terms of reducing mortality risk, severe cardiometabolic events, and general morbidity, particularly injuries, should be determined [112]. This assessment could occur in the form of a registry study in which adverse events due to defined training forms are systematically recorded, including the long-term effects of myocardial abnormalities and conduction disorders. The Sudden Cardiac Death Register in Germany [113, 114] can serve as a foundation for this as it provides valuable data on the incidence, risk factors, and preventive measures related to sudden cardiac death, particularly in athletes.

In addition, a national cohort of healthy individuals should be established to determine comparative benchmarks for cardiopulmonary fitness, modeled, for example, on the US FRIEND Registry but expanded to include aspects of muscular fitness [94]. It would be desirable to examine the extent to which the inclusion of cardiorespiratory and/or muscular fitness in the above scores can contribute to better risk assessment results. Finally, the extent to which this structured approach can help motivate more individuals to engage in physical activity should be examined.

## Conclusions

This consensus-based guideline was developed to provide a strong foundation for evidence-informed PPEs. Its recommendations are intended to guide (sports medicine) physicians in identifying individuals who are at risk of injury or fatal events before they participate in relatively intense physical activity. If abnormalities are found, the appropriate specialists should be consulted (e.g., orthopedists and sports cardiologists). The results of these examinations can also be used to document an individual’s health status and facilitate appropriate counseling consistent with the FITT-VP principle on the basis of MI. Future studies should examine this guideline’s feasibility in various regions, including resource-limited settings, and the extent to which it prevents harmful or potentially fatal events. On the basis of this, appropriate adjustments should be implemented, considering the integration of the sports medicine specialty across Europe.

## Supplementary Information

Below is the link to the electronic supplementary material.

Supplementary file1 (DOCX 133 KB)

## Abbreviations

ACSM: American College of Sports Medicine
AWMF: Association of Scientific Medical Societies in Germany (Arbeitsgemeinschaft der Wissenschaftlichen Medizinischen Fachgesellschaften)
Arriba: Define the task jointly, subjective risk, objective risk, information about prevention options, assessing the options, agreeing on how to proceed (Aufgabe gemeinsam definieren, Risiko subjektiv, Risiko objektiv, Information über Präventionsmöglichkeiten, Bewertung der Möglichkeiten, Absprache über weiteres Vorgehen)
CHD: Coronary heart disease
COPD: Chronic obstructive pulmonary disease
COVID-19: Coronavirus disease-2019
CPET: Cardiopulmonary exercise testing
DEGAM: German Society for General Practice and Family Medicine (Deutsche Gesellschaft für Allgemeinmedizin und Familienmedizin)
DGSP: German Society of Sports Medicine and Prevention (Deutsche Gesellschaft für Sportmedizin und Prävention)
ECG: Electrocardiogram
EFSMA: European Federation of Sports Medicine Associations
EU: European Union
IFOM: Institute for Research in Operative Medicine
MET: Metabolic equivalent
NATA: National Athletic Trainers’ Association
PAD: Peripheral artery disease
PPE: Sports preparticipation evaluation
RED-S: Relative energy deficit in sport
SARS-CoV-2: Acute respiratory syndrome-coronavirus-2
TTE: Transthoracic echocardiography
USA: United States
WHO: World Health Organization

## Declarations

### Data availability

The data presented in this study are available on reasonable request from the corresponding author.
