# Supplementary material for: Sports Preparticipation Evaluation for Healthy Adults: A Consensus-Based German Guideline
Source: Sports Med. 2025 Jul 3;55(8):1827–51. doi: 10.1007/s40279-025-02230-5 (PMC12460515; doi:10.1007/s40279-025-02230-5)
Supplement: Supplementary file 1 — Supplementary file1 (DOCX 133 KB) [file 40279_2025_2230_MOESM1_ESM.docx]

# Supplementary material

**SPORTS MEDICINE**

**Sports pre-participation evaluation for healthy adults: A consensus-based German guideline**

Short title:

Consensus-Based German Guideline on Sports Pre-Participation Evaluation

Christine Joisten*^1,2^, Anja Hirschmüller*^2,3,4^ Pascal Bauer^5,6^, Erika Baum^7,8^, Meinolf Behrens^9,10^, Susanne Berrisch-Rahmel^6,11^, Gregor Berrsche^12,13^, Anja Carlsohn^2,14^, Michael Cassel^2,15^, Justus DeZeuuw^16,17^, Gesine Dörr^18,19^, Michael Dreher^17,20^, Frank Edelmann^21,22^, Katrin Esefeld^6,23^, Michael Freitag^8,24^, Mathias Grebe^19,25^, Casper Grim^26,27^, Pia Janßen^2,28^, Rolf Kaiser^29,30^ , Thomas Katlun^2,31^, Maximilian Köppel^32,33^, Charlotte Kreutz^2^, Karsten Krüger^2,34^, Christoph Lutter^13,35^, Frank Mayer^2,36^, Othmar Moser^10,37^, Andreas Nieß^2,28^, Hans-Georg Predel^38,39^ , Stefan Peters^33,40^, Petra Platen^2,41^, Dorothea Portius^42,43^ , Claus Reinsberger^2,44^, Nils Reiss^45,46^ , Kai Röcker^2,47^, Thomas Schmidt^38,46^, Arno Schmidt-Trucksäss^2,48^, Holger Schmitt^2,12^, Thomas Schramm^2,49^, Christian Sturm^50,51^ , Hans Vater^30,52^, Alina Weise^53^, Burkhard Weisser^39,54^, Götz Welsch^55,56^, Andreas Winkelmann^51,57^, Alfred Wirth^43,58^, Bernd Wolfarth^2,27,59^, Käthe Goossen^53^

* shared first authorship

Emailadresses of the corresponding authors

Prof. Dr. Dr. Christine Joisten

[c.joisten@dshs-koeln.de](mailto:c.joisten@dshs-koeln.de)

and

Prof. Dr. Anja Hirschmüller

[anja.hirschmueller@altius.ag](mailto:anja.hirschmueller@altius.ag)

**Affiliations**

1. German Sport University Cologne, Institute of Movement and Neurosciences, Cologne, Germany.
2. German Society of Sports Medicine and Prevention, Frankfurt a.M., Germany.
3. ALTIUS Swiss Sportmed Center, Rheinfelden, Switzerland.
4. Department of Orthopedics and Trauma Surgery, Medical Center, Albert-Ludwigs-University of Freiburg, Freiburg, Germany
5. Department of Cardiology and Angiology, Justus Liebig University Giessen, Germany.
6. German Society for Cardiology – Cardiovascular Research (DGK), Germany.
7. Department of General Practice, University of Marburg, Germany.
8. German Society for General Practice and Family Medicine (DEGAM), Germany.
9. Diabetes Centre Minden, Minden, Germany.
10. German Diabetes Society (DDG), Working Group Diabetes, Sport, and Exercise. Germany.
11. KardioPro, Practice for Internal Medicine, Cardiology, Sports Medicine, and Sports Cardiology, Düsseldorf, Germany.
12. Centre for Orthopaedics and Sports Trauma Surgery, Atos Clinic Heidelberg, Germany.
13. Society for Orthopaedic-Traumatological Sports Medicine (GOTS), Germany.
14. Department of Nutrition and Home Economics, University of Applied Sciences Hamburg, Hamburg, Germany.
15. University of Potsdam, Faculty of Health Sciences, Potsdam, Brandenburg, Germany.
16. Healthy Heart MVZ in Cologne, Cologne, Germany.
17. German Society for Pneumology and Respiratory Medicine (DGP), Germany.
18. Alexianer St. Josefs Hospital Potsdam-Sanssouci, Potsdam, Germany.
19. German Society for Angiology – Society for Vascular Medicine (DGA), Germany.
20. Department of Pneumology and Intensive Care Medicine, University Hospital RWTH Aachen, Aachen, Germany.
21. Department of Internal Medicine and Cardiology, Campus Virchow Hospital, Charité University Medicine Berlin, Berlin, Germany.
22. German Society for Internal Medicine (DGIM), Germany.
23. Department of Prevention and Sports Medicine, University Hospital Klinikum rechts der Isar, Technical University of Munich, Munich, Germany.
24. Department of General Medicine, Department of Health Services Research, Carl von Ossietzky University Oldenburg, Oldenburg, Germany.
25. Centre of Cardiac and Vascular Diseases, Marburg, Germany.
26. Centre for Musculoskeletal Surgery Osnabrück, Osnabrück Hospital, Osnabrück, Germany.
27. German Olympic Sports Confederation (DOSB), Germany.
28. Department of Sports Medicine, University Hospital of Tübingen, Tübingen, Germany.
29. Clinic for Internal Medicine I, Cardiology, Angiology, Diabetology and Sports Medicine, Hanse and University City of Rostock, Rostock, Germany.
30. German Disabled Sports Association and National Paralympic Committee e.V. (DBS), Germany.
31. Katlun Eye Clinic, Heidelberg, Germany.
32. Working Group Oncological Sports and Exercise Therapy, National Centre for Tumour Diseases Heidelberg, Heidelberg, Germany.
33. German Association for Health-Related Physical Activity and Exercise Therapy (DVGS), Hürth, Germany.
34. Department of Exercise Physiology and Sports Therapy, Institute of Sports Science, Giessen, Germany.
35. Department of Orthopaedics, Rostock University Medical Center, Rostock, Germany.
36. University of Potsdam, Centre of Sports Medicine, University Outpatient Clinic, Potsdam, Germany.
37. Exercise Physiology & Metabolism (Sports Medicine), Bayreuth Centre of Sports Science, University of Bayreuth, Bayreuth, Germany.
38. German Sport University Cologne, Institute of Cardiology and Sports Medicine, Cologne, Germany.
39. German Hypertension League (DHL), Germany.
40. Department of Human Sciences, Institute of Sport Science, Bundeswehr University Munich, Neubiberg, Germany
41. Department of Sports Medicine and Sports Nutrition, Faculty of Sports Science, Ruhr University Bochum, Bochum, Germany.
42. Martin Luther University Halle-Wittenberg, Institute of Agricultural and Nutritional Sciences, Halle, Germany.
43. German Obesity Society (DAG), Germany.
44. Institute of Sports Medicine, Paderborn University, Paderborn, Germany.
45. Schüchtermann Clinic Bad Rothenfelde, Bad Rothenfelde, Germany.
46. German Society for Prevention and Rehabilitation of Cardiovascular Diseases (DGPR), Germany.
47. Institute for Applied Health Promotion and Exercise (IFAG), Furtwangen University, Furtwangen, Germany.
48. University of Basel, Sport and Exercise Medicine, Department of Sport, Exercise and Health, Basel, Switzerland.
49. Cardiology Rodenkirchen, Cologne, Germany.
50. Department of Rehabilitation and Sports Medicine, Hannover Medical School, Hannover, Germany
51. German Society for Physical and Rehabilitative Medicine (DGPRM), Germany.
52. Prof. Vater & Colleagues, Bad Wildungen, Germany.
53. Witten/Herdecke University, Institute for Research in Operative Medicine (IFOM), Cologne, Germany.
54. Institute for Sports Science, Christian-Albrechts-University Kiel, Kiel, Germany.
55. Athleticum, Department of Sports Medicine and Department of Trauma and Orthopedic Surgery at University Medical Center Hamburg-Eppendorf (UKE), Germany.
56. German Society for Orthopaedics and Trauma Surgery (DGOU), Germany.
57. Department of Orthopaedics and Trauma Surgery, Musculoskeletal University Center Munich (MUM), LMU University Hospital, LMU Munich, Germany.
58. Teutoburger Wald Clinic, Bad Rothenfelde, Germany.
59. Department of Sports Medicine, Humboldt University and Charité University School of Medicine, Berlin, Germany.

# Suppl. 1 Evaluation of possible conflicts of interest

The following topic-related conflicts of interest were identified by the conflict-of-interest officers:

- **Minor, direct conflict of interest: Restriction of management function (coordination/AG management)**

Lecture fees (industry, especially manufacturers/developers of devices or food supplements)

- **Minor, indirect conflict of interest: Request to abstain from voting on thematically relevant recommendations**

Leading position in regional sports medicine associations or the DGSP (thematically relevant for recommendation 6)

- **Moderate conflict of interest: Abstention from voting on thematically relevant recommendations**

Expert/consultant activities outside of advisory boards (industry, especially manufacturers/developers of devices or dietary supplements)

Advisory board activities (only thematically relevant for health insurance companies if reimbursement decisions were made in the corresponding committee)

Leading function in third-party-funded research for industry/companies

- **High conflict of interest: No co-authorship of thematically relevant sections;**

**abstention from voting on thematically relevant recommendations**

Company ownership

Patents on medical devices

Research with public funds only acquired or managed as applicants was recorded;

however, this does not primarily represent a conflict of interest.

# Suppl. 2 Characteristics and quality appraisal of included documents (see [13] and reference list)

The complete results of the guideline synopsis can be found in the guideline report and in Weise et al. [13].

*Strength of the recommendations*

A total of 12.8% (39/305) of the recommendations were directly linked to evidence from 55 primary studies. The level of evidence (LoE) for these primary studies was distributed as follows: 3.6% (2/55) LoE1, 21.8% (12/55) LoE2, 32.7% (18/55) LoE3, and 41.8% (23/55) LoE4. In 266 of the 305 recommendations (87.2%), there was no direct reference to evidence from primary studies. The strength of the recommendations, according to the SORT taxonomy, was A for 1.3% (4/305), B for 4.6% (14/305) and C for 24.3% (74/305) of them. Of the 305 recommendations, 213 (69.8%) not explicitly labelled as recommendations by the authors (Figure S1).

Figure S1: Basis for recommendations from the included guidelines and consensus documents (% of total recommendations in guidelines/consensus documents)

Table S1: Characteristics and quality appraisal of included documents Weise et al. [13].

| **ID** | **Title** | **Region, year** | **Population** | **AGREE-II, Domain:** | |
| --- | --- | --- | --- | --- | --- |
| **Ref.** | **Organisation** |  |  |  |  |
|  |  |  |  | **3** | **6** |
| AAFP 2016 [1] | *Selected Issues in Injury and Illness Prevention and the Team Physician: A Consensus Statement* | USA, 2016 | athletes | 4% | 0% |
|  | unclear (several) |  |  |  |  |
| AAFP 2017 [2] | *Female Athlete Issues for the Team Physician: A Consensus Statement - 2017 Update* | USA, 2017 | female athletes, pregnant athletes | 7% | 0% |
|  | American Academy for Family Physicians |  |  |  |  |
| AAP 2019 [3] | *Preparticipation Physical Evaluation, 5th Edition* | USA, 2019 | athletes in organised sports or vigorous physical activities | 14% | 0% |
|  | American Academy of Pediatrics |  |  |  |  |
| ACOG 2020 [4] | *Physical Activity and Exercise During Pregnancy and the Postpartum Period: ACOG Committee Opinion, Number 804* | USA, 2020 | pregnant women | 7% | 17% |
|  | American College of Obstetricians and Gynecologists |  |  |  |  |
| ACPM 2013 [5] | *Screening for sudden cardiac death before participation in high school and collegiate sports: American College of Preventive Medicine position statement on preventive practice* | USA, 2013 | high school and college athletes | 10% | 25% |
|  | American College of Preventive Medicine |  |  |  |  |
| ACSM 2019 [6] | *Exercise Guidelines for Cancer Survivors: Consensus Statement from International Multidisciplinary Roundtable* | USA, 2019 | cancer survivors | 24% | 50% |
|  | American College of Sports Medicine |  |  |  |  |
| ACSM 2021 [7] | *ACSM’s Guidelines for Exercise Testing and Prescription, 11th Edition* | USA, 2021 | general population (incl. pregnant women and cancer survivors) | 11% | 0% |
|  | American College of Sports Medicine |  |  |  |  |
| AEPC 2017 [8] | *Cardiovascular pre-participation screening in young athletes: Recommendations of the Association of European Paediatric Cardiology* | Europe, 2017 | young athletes | 13% | 75% |
|  | Association of European Paediatric Cardiology |  |  |  |  |
| AHA ACC 2015 [9-11] | *Eligibility and Disqualification Recommendations for Competitive Athletes With Cardiovascular Abnormalities:* | USA, 2015 | general population, participants in organised sports, athletes | 41% | 33% |
|  | *Preamble, Principles, and General Considerations; Task Force 2: Preparticipation Screening for Cardiovascular Disease in Competitive Athletes; Task Force 6: Hypertension:* |  |  |  |  |
|  | *A Scientific Statement From the American Heart Association and American College of Cardiology* |  |  |  |  |
|  | American Heart Association, American College of Cardiology |  |  |  |  |
| AMSSM 2017 [12] | *AMSSM Position Statement on Cardiovascular Preparticipation Screening in Athletes: Current evidence, knowledge gaps, recommendations and future directions* | USA, 2017 | athletes | 20% | 25% |
|  | American Medical Society for Sports Medicine |  |  |  |  |
| AMSSM 2017 (ECG) [13] | *International criteria for electrocardiographic interpretation in athletes: Consensus statement* | World, 2017 | athletes | 20% | 46% |
|  | unklar (mehrere) |  |  |  |  |
| AMSSM 2020 [14] | *Mental health issues and psychological factors in athletes: detection, management, effect on performance and prevention: American Medical Society for Sports Medicine Position Statement-Executive Summary* | USA, 2020 | athletes | 24% | 71% |
|  | American Medical Society for Sports Medicine |  |  |  |  |
| ASE 2020 [15] | *Recommendations on the Use of Multimodality Cardiovascular Imaging in Young Adult Competitive Athletes: A Report from the American Society of Echocardiography in Collaboration with the Society of Cardiovascular Computed Tomography and the Society for Cardiovascular Magnetic Resonance* | USA, 2020 | young athletes | 19% | 21% |
|  | American Society of Echocardiography |  |  |  |  |
| BSE CRY 2018 [16] | *A guideline update for the practice of echocardiography in the cardiac screening of sports participants: a joint policy statement from the British Society of Echocardiography and Cardiac Risk in the Young* | UK, 2018 | young athletes | 9% | 71% |
|  | British Society of Echocardiography and Cardiac Risk in the Young |  |  |  |  |
| CASEM 2020 [17] | *Physical activity prescription: a critical opportunity to address a modifiable risk factor for the prevention and management of chronic disease: a position statement by the Canadian Academy of Sport and Exercise Medicine* | World, 2020 | general population | 8% | 21% |
|  | Canadian Academy of Sport and Exercise Medicine |  |  |  |  |
| CCS CHRS 2019 [18] | *Canadian Cardiovascular Society/Canadian Heart Rhythm Society Joint Position Statement on the Cardiovascular Screening of Competitive Athletes* | Canada, 2019 | athletes | 42% | 54% |
|  | Canadian Cardiovascular Society, Canadian Heart Rhythm Society |  |  |  |  |
| COCIS 2021 [19-21] | *Italian cardiological guidelines for sports eligibility in athletes with heart disease: part 1; part 2; Italian Cardiological Guidelines (COCIS) for Competitive Sport Eligibility in athletes with heart disease: update 2020* | Italy, 2021 | athletes | 8% | 0% |
|  | Italian Society of Sports Cardiology and the Italian Sports Medicine Federation |  |  |  |  |
| EA4SD 2020 [22] | *The European Association for Sports Dentistry, Academy for Sports Dentistry, European College of Sports and Exercise Physicians consensus statement on sports dentistry integration in sports medicine* | Europe/ USA, 2020 | athletes of all levels | 7% | 21% |
|  | European Association for Sports Dentistry |  |  |  |  |
| EAPC EACVI 2018 [23, 24] | *The multi-modality cardiac imaging approach to the Athlete's heart: an expert consensus of the European Association of Cardiovascular Imaging; European Association of Preventive Cardiology (EAPC) and European Association of Cardiovascular Imaging (EACVI) joint position statement: recommendations for the indication and interpretation of cardiovascular imaging in the evaluation of the athlete's heart* | Europe, 2018 | athletes and elite athletes | 10% | 21% |
|  | European Association of Cardiovascular Imaging |  |  |  |  |
| EFSMA 2015 [25] | *The Pre-Participation Examination in Sports: EFSMA Statement on ECG for Pre-Participation Examination* | Europe, 2015 | recreational to elite athletes | 14% | 21% |
|  | European Federation of Sports Medicine Associations |  |  |  |  |
| EFSMA 2021 [26] | *Preparticipation medical evaluation for elite athletes: EFSMA recommendations on standardised preparticipation evaluation form in European countries* | Europe, 2021 | elite athletes | 8% | 33% |
|  | European Federation of Sports Medicine Associations |  |  |  |  |
| EHRA EACPR 2017 [27] | *Pre-participation cardiovascular evaluation for athletic participants to prevent sudden death: Position paper from the EHRA and the EACPR, branches of the ESC. Endorsed by APHRS, HRS, and SOLAECE* | Europe, 2017 | athletes | 14% | 13% |
|  | European Heart Rhythm Association, European Association for Cardiovascular Prevention and Rehabilitation |  |  |  |  |
| ESC 2021 [28] | *2020 ESC Guidelines on sports cardiology and exercise in patients with cardiovascular disease* | Europe, 2021 | general population (incl. Cancer survivors) and athletes | 41% | 63% |
|  | European Society of Cardiology |  |  |  |  |
| ESC 2022 [29] | *2022 ESC Guidelines for the management of patients with ventricular arrhythmias and the prevention of sudden cardiac death* | Europe, 2022 | middle aged and elderly individuals and athletes | 32% | 75% |
|  | European Society of Cardiology |  |  |  |  |
| FATC 2014 [30] | *2014 Female Athlete Triad Coalition Consensus Statement on Treatment and Return to Play of the Female Athlete Triad* | USA, 2014 | female athletes | 15% | 21% |
|  | Female Athlete Triad Coalition |  |  |  |  |
| FMATC 2021 [31, 32] | *The Male Athlete Triad-A Consensus Statement From the Female and Male Athlete Triad Coalition Part 1: Definition and Scientific Basis; Part II: Diagnosis, Treatment, and Return-To-Play* | USA, 2021 | male athletes | 14% | 21% |
|  | Female and Male Athlete Triad Coalition |  |  |  |  |
| FSC 2019 [33, 34] | *French Society of Cardiology guidelines on exercise tests (part 1): Methods and interpretation; (part 2): Indications for exercise tests in cardiac diseases* | France, 2019 | athletes | 17% | 71% |
|  | French Society of Cardiology |  |  |  |  |
| IOC 2013 [35] | *How to minimise the health risks to athletes who compete in weight-sensitive sports review and position statement on behalf of the Ad Hoc Research Working Group on Body Composition, Health and Performance, under the auspices of the IOC Medical Commission* | World, 2013 | athletes in weight-sensitive sports | 7% | 42% |
|  | International Olympic Committee |  |  |  |  |
| IOC 2017 [36] | *Exercise and pregnancy in recreational and elite athletes: 2016/2017 evidence summary from the IOC expert group meeting, Lausanne. Part 5. Recommendations for health professionals and active women* | World, 2017 | pregnant and post-partum recreational and elite athletes | 6% | 75% |
|  | International Olympic Committee |  |  |  |  |
| IOC 2018 [37, 38] | *The IOC consensus statement: beyond the Female Athlete Triad--Relative Energy Deficiency in Sport (RED-S); International Olympic Committee (IOC) Consensus Statement on Relative Energy Deficiency in Sport (RED-S): 2018 Update* | World, 2018 | athletes | 7% | 42% |
|  | International Olympic Committee |  |  |  |  |
| NATA 2012 [39] | *National athletic trainers' association position statement: preventing sudden death in sports* | USA, 2012 | participants in organised sports | 15% | 0% |
|  | National Athletic Trainers’ Association |  |  |  |  |
| NATA 2013 [40] | *The inter-association task force for preventing sudden death in secondary school athletics programs: best-practices recommendations* | North America, 2013 | secondary school athletes | 8% | 0% |
|  | unklar (mehrere) |  |  |  |  |
| NATA 2014 [41] | *National Athletic Trainers' Association position statement: Preparticipation physical examinations and disqualifying conditions* | USA, 2014 | participants in organised sports | 17% | 0% |
|  | National Athletic Trainers’ Association |  |  |  |  |
| NATA 2015 [42] | *Interassociation recommendations for developing a plan to recognize and refer student-athletes with psychological concerns at the secondary school level: a consensus statement* | USA, 2015 | secondary school athletes | 13% | 0% |
|  | Unclear (several) |  |  |  |  |
| NCAA 2016 [43] | *Interassociation Consensus Statement on Cardiovascular Care of College Student-Athletes* | USA, 2016 | college athletes | 10% | 42% |
|  | National Collegiate Athletic Association |  |  |  |  |

References Table S2

1. Selected Issues in Injury and Illness Prevention and the Team Physician: A Consensus Statement. Curr Sports Med Rep. 2016;15(1):48-59.

2. Female Athlete Issues for the Team Physician: A Consensus Statement - 2017 Update. Medicine and Science in Sports and Exercise. 2018;50(5):1113-22.

3. American Academy of Pediatrics, American Academy of Family Physicians, American College of Sports Medicine, American Medical Society for Sports Medicine, American Orthopaedic Society for Sports Medicine, American Osteopathic Academy of Sports Medicine. Preparticipation Physical Evaluation, 5th Edition: American Academy of Pediatrics; 2019. 240 p.

4. Physical Activity and Exercise During Pregnancy and the Postpartum Period: ACOG Committee Opinion, Number 804. Obstet Gynecol. 2020;135(4):e178-e88.

5. Mahmood S, Lim L, Akram Y, Alford-Morales S, Sherin K. Screening for sudden cardiac death before participation in high school and collegiate sports: American College of Preventive Medicine position statement on preventive practice. Am J Prev Med. 2013;45(1):130-3.

6. Campbell KL, Winters-Stone KM, Wiskemann J, May AM, Schwartz AL, Courneya KS, et al. Exercise Guidelines for Cancer Survivors: Consensus Statement from International Multidisciplinary Roundtable. Med Sci Sports Exerc. 2019;51(11):2375-90.

7. American College of Sports Medicine. ACSM’s Guidelines for Exercise Testing and Prescription, 11th Edition. 11th ed2021.

8. Fritsch P, Ehringer-Schetitska D, Dalla Pozza R, Jokinen E, Herceg-Cavrak V, Hidvegi E, et al. Cardiovascular pre-participation screening in young athletes: Recommendations of the Association of European Paediatric Cardiology. Cardiol Young. 2017;27(9):1655-60.

9. Maron BJ, Levine BD, Washington RL, Baggish AL, Kovacs RJ, Maron MS. Eligibility and Disqualification Recommendations for Competitive Athletes With Cardiovascular Abnormalities: Task Force 2: Preparticipation Screening for Cardiovascular Disease in Competitive Athletes: A Scientific Statement From the American Heart Association and American College of Cardiology. Circulation. 2015;132(22):e267-72.

10. Maron BJ, Zipes DP, Kovacs RJ. Eligibility and Disqualification Recommendations for Competitive Athletes With Cardiovascular Abnormalities: Preamble, Principles, and General Considerations: A Scientific Statement From the American Heart Association and American College of Cardiology. Circulation. 2015;132(22):e256-61.

11. Black HR, Sica D, Ferdin K, White WB. Eligibility and Disqualification Recommendations for Competitive Athletes With Cardiovascular Abnormalities: Task Force 6: Hypertension: A Scientific Statement from the American Heart Association and the American College of Cardiology. Circulation. 2015;132(22):e298-302.

12. Drezner JA, O'Connor FG, Harmon KG, Fields KB, Asplund CA, Asif IM, et al. AMSSM Position Statement on Cardiovascular Preparticipation Screening in Athletes: Current evidence, knowledge gaps, recommendations and future directions. Br J Sports Med. 2017;51(3):153-67.

13. Drezner JA, Sharma S, Baggish A, Papadakis M, Wilson MG, Prutkin JM, et al. International criteria for electrocardiographic interpretation in athletes: Consensus statement. Br J Sports Med. 2017;51(9):704-31.

14. Chang C, Putukian M, Aerni G, Diamond A, Hong G, Ingram Y, et al. Mental health issues and psychological factors in athletes: detection, management, effect on performance and prevention: American Medical Society for Sports Medicine Position Statement-Executive Summary. Br J Sports Med. 2020;54(4):216-20.

15. Baggish AL, Battle RW, Beaver TA, Border WL, Douglas PS, Kramer CM, et al. Recommendations on the Use of Multimodality Cardiovascular Imaging in Young Adult Competitive Athletes: A Report from the American Society of Echocardiography in Collaboration with the Society of Cardiovascular Computed Tomography and the Society for Cardiovascular Magnetic Resonance. J Am Soc Echocardiogr. 2020;33(5):523-49.

16. Oxborough D, Augustine D, Gati S, George K, Harkness A, Mathew T, et al. A guideline update for the practice of echocardiography in the cardiac screening of sports participants: a joint policy statement from the British Society of Echocardiography and Cardiac Risk in the Young. Echo Res Pract. 2018;5(1):G1-g10.

17. Thornton JS, Frémont P, Khan K, Poirier P, Fowles J, Wells GD, et al. Physical Activity Prescription: A Critical Opportunity to Address a Modifiable Risk Factor for the Prevention and Management of Chronic Disease: A Position Statement by the Canadian Academy of Sport and Exercise Medicine: Erratum. Clin J Sport Med. 2020;30(6):616.

18. Johri AM, Poirier P, Dorian P, Fournier A, Goodman JM, McKinney J, et al. Canadian Cardiovascular Society/Canadian Heart Rhythm Society Joint Position Statement on the Cardiovascular Screening of Competitive Athletes. Can J Cardiol. 2019;35(1):1-11.

19. Delise P, Mos L, Sciarra L, Basso C, Biffi A, Cecchi F, et al. Italian Cardiological Guidelines (COCIS) for Competitive Sport Eligibility in athletes with heart disease: update 2020. J Cardiovasc Med (Hagerstown). 2021;22(11):874-91.

20. Biffi A, Delise P, Zeppilli P, Giada F, Pelliccia A, Penco M, et al. Italian cardiological guidelines for sports eligibility in athletes with heart disease: part 1. J Cardiovasc Med (Hagerstown). 2013;14(7):477-99.

21. Biffi A, Delise P, Zeppilli P, Giada F, Pelliccia A, Penco M, et al. Italian cardiological guidelines for sports eligibility in athletes with heart disease: part 2. J Cardiovasc Med (Hagerstown). 2013;14(7):500-15.

22. Stamos A, Mills S, Malliaropoulos N, Cantamessa S, Dartevelle JL, Gündüz E, et al. The European Association for Sports Dentistry, Academy for Sports Dentistry, European College of Sports and Exercise Physicians consensus statement on sports dentistry integration in sports medicine. Dent Traumatol. 2020;36(6):680-4.

23. Galderisi M, Cardim N, D'Andrea A, Bruder O, Cosyns B, Davin L, et al. The multi-modality cardiac imaging approach to the Athlete's heart: an expert consensus of the European Association of Cardiovascular Imaging. Eur Heart J Cardiovasc Imaging. 2015;16(4):353.

24. Pelliccia A, Caselli S, Sharma S, Basso C, Bax JJ, Corrado D, et al. European Association of Preventive Cardiology (EAPC) and European Association of Cardiovascular Imaging (EACVI) joint position statement: recommendations for the indication and interpretation of cardiovascular imaging in the evaluation of the athlete's heart. Eur Heart J. 2018;39(21):1949-69.

25. Löllgen H, Börjesson M, Cummiskey J, Bachl N, Debruyne A. The Pre-Participation Examination in Sports: EFSMA Statement on ECG for Pre-Participation Examination. Deutsche Zeitschrift für Sportmedizin. 2015;66(6):151-5 doi.

26. Ionescu AM, Pitsiladis YP, Rozenstoka S, Bigard X, Löllgen H, Bachl N, et al. Preparticipation medical evaluation for elite athletes: EFSMA recommendations on standardised preparticipation evaluation form in European countries. BMJ Open Sport Exerc Med. 2021;7(4):e001178.

27. Mont L, Pelliccia A, Sharma S, Biffi A, Borjesson M, Brugada Terradellas J, et al. Pre-participation cardiovascular evaluation for athletic participants to prevent sudden death: Position paper from the EHRA and the EACPR, branches of the ESC. Endorsed by APHRS, HRS, and SOLAECE. Eur J Prev Cardiol. 2017;24(1):41-69.

28. Pelliccia A, Sharma S, Gati S, Bäck M, Börjesson M, Caselli S, et al. 2020 ESC Guidelines on sports cardiology and exercise in patients with cardiovascular disease. European Heart Journal. 2021;42(1):17-96.

29. Zeppenfeld K, Tfelt-Hansen J, de Riva M, Winkel BG, Behr ER, Blom NA, et al. 2022 ESC Guidelines for the management of patients with ventricular arrhythmias and the prevention of sudden cardiac death. Eur Heart J. 2022;43(40):3997-4126.

30. De Souza MJ, Nattiv A, Joy E, Misra M, Williams NI, Mallinson RJ, et al. 2014 Female Athlete Triad Coalition Consensus Statement on Treatment and Return to Play of the Female Athlete Triad: 1st International Conference held in San Francisco, California, May 2012 and 2nd International Conference held in Indianapolis, Indiana, May 2013. Br J Sports Med. 2014;48(4):289.

31. Fredericson M, Kussman A, Misra M, Barrack MT, De Souza MJ, Kraus E, et al. The Male Athlete Triad-A Consensus Statement From the Female and Male Athlete Triad Coalition Part II: Diagnosis, Treatment, and Return-To-Play. Clin J Sport Med. 2021;31(4):349-66.

32. Nattiv A, De Souza MJ, Koltun KJ, Misra M, Kussman A, Williams NI, et al. The Male Athlete Triad-A Consensus Statement From the Female and Male Athlete Triad Coalition Part 1: Definition and Scientific Basis. Clin J Sport Med. 2021;31(4):335–48.

33. Marcadet DM, Pavy B, Bosser G, Claudot F, Corone S, Douard H, et al. French Society of Cardiology guidelines on exercise tests (part 1): Methods and interpretation. Arch Cardiovasc Dis. 2018;111(12):782-90.

34. Marcadet DM, Pavy B, Bosser G, Claudot F, Corone S, Douard H, et al. French Society of Cardiology guidelines on exercise tests (part 2): Indications for exercise tests in cardiac diseases. Arch Cardiovasc Dis. 2019;112(1):56-66.

35. Sundgot-Borgen J, Meyer NL, Lohman TG, Ackl TR, Maughan RJ, Stewart AD, et al. How to minimise the health risks to athletes who compete in weight-sensitive sports review and position statement on behalf of the Ad Hoc Research Working Group on Body Composition, Health and Performance, under the auspices of the IOC Medical Commission. Br J Sports Med. 2013;47(16):1012-22.

36. Bø K, Artal R, Barakat R, Brown WJ, Davies GAL, Dooley M, et al. Exercise and pregnancy in recreational and elite athletes: 2016/2017 evidence summary from the IOC expert group meeting, Lausanne. Part 5. Recommendations for health professionals and active women. Br J Sports Med. 2018;52(17):1080-5.

37. Mountjoy M, Sundgot-Borgen J, Burke L, Ackerman KE, Blauwet C, Constantini N, et al. International Olympic Committee (IOC) Consensus Statement on Relative Energy Deficiency in Sport (RED-S): 2018 Update. Int J Sport Nutr Exerc Metab. 2018;28(4):316-31.

38. Mountjoy M, Sundgot-Borgen J, Burke L, Carter S, Constantini N, Lebrun C, et al. The IOC consensus statement: beyond the Female Athlete Triad--Relative Energy Deficiency in Sport (RED-S). Br J Sports Med. 2014;48(7):491-7.

39. Casa DJ, Guskiewicz KM, Anderson SA, Courson RW, Heck JF, Jimenez CC, et al. National athletic trainers' association position statement: preventing sudden death in sports. J Athl Train. 2012;47(1):96-118.

40. Casa DJ, Almquist J, Anderson SA, Baker L, Bergeron MF, Biagioli B, et al. The inter-association task force for preventing sudden death in secondary school athletics programs: best-practices recommendations. J Athl Train. 2013;48(4):546-53.

41. Conley KM, Bolin DJ, Carek PJ, Konin JG, Neal TL, Violette D. National Athletic Trainers' Association position statement: Preparticipation physical examinations and disqualifying conditions. J Athl Train. 2014;49(1):102-20.

42. Neal TL, Diamond AB, Goldman S, Liedtka KD, Mathis K, Morse ED, et al. Interassociation recommendations for developing a plan to recognize and refer student-athletes with psychological concerns at the secondary school level: a consensus statement. J Athl Train. 2015;50(3):231-49.

43. Hainline B, Drezner J, Baggish A, Harmon KG, Emery MS, Myerburg RJ, et al. Interassociation Consensus Statement on Cardiovascular Care of College Student-Athletes. J Athl Train. 2016;51(4):344-57.

# Suppl. 3 Medical history and examination forms


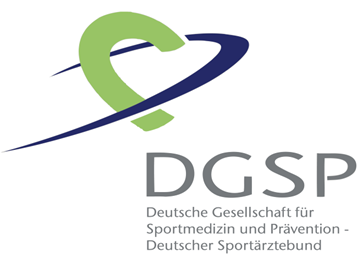


PATIENT LABEL

**Medical history**

**Date: Name: Date of birth:**

**1. What is the reason for your visit?**

sports newcomer  returning to sports  regular sports activity

**2. Training and activity anamnesis**

Please complete the table even if you do not participate in a sport. At the very least, you should provide information on everyday activities; information on walking activities lasting more than 5 minutes is necessary (see the example table). Please note the following when completing the table: You are asked about your average activity behavior **over the last three months**. Differentiate training and everyday activities, and for each activity, also state the perceived intensity of exertion (L: Light, M: Moderate, I: Intensive; see the table’s footnote). If you warm up or down as part of mixed training (e.g., in the gym, strength training) with endurance exercises, please enter the weekly duration in minutes in the last line marked.

| Type of sport/training | Number of training sessions per week | Total amount per week in minutes | Exercise intensity* | Since when? (year) |
| --- | --- | --- | --- | --- |
|  |  |  |  |  |
|  |  |  |  |  |
|  |  |  |  |  |
| Endurance exercise for warming up and warming down as part of the above training program |  |  |  |  |
| Physical activity in everyday life | Frequency per week | Total amount per week in minutes | Exercise intensity |  |
| Walking longer than 5 minutes |  |  |  |  |
|  |  |  |  |  |
| Sitting time at work |  |  |  |  |
| Sitting time in everyday life |  |  |  |  |

* Exercise intensity:

**Light (L):** Calm breathing, hardly any sweating (e.g., walking calmly on level ground)

**Moderate (M):** Beginning increased breathing, fluent speech still possible, some sweating

(e.g., brisk walking, easy jogging, biking on the flat, slow swimming, moderate gardening)

**Intensive (I):** Increased breathing, talking only possible to a limited extent, increased sweating

(e.g., endurance running, interval training, brisk biking and/or biking on rough terrain, sports such as tennis or football, heavy gardening)

2.1. When was the last time you trained?

today  yesterday  2 days ago  >2 days ago

2.2. If you participate in sports, how would you categorize them?

leisure sports  ambitious popular sports  competitive sports

**Recreational sports** are sporting activities without or with only a low-performance aspect and/or health sports training;

**Ambitious popular sports** include sports training that aims to improve performance and includes at least occasional performance of competition-like activities;

**Competitive sports** generally involve daily training, a clear focus on competitive success, and organization in a club or sports association.

2.3. Did you used to take part in competitive sports?

yes  no if yes:  ambitious popular sports  competitive sports

Up to what age? ____ years old Level (e.g. Bundesliga, squad)? _____________________

**3. Do you have any current complaints?**   yes  no

3.1. If yes, which ________________________________________________________________________

3.2. Do symptoms occur specifically during physical exertion/sports?  yes  no

3.3. If so, what complaints have you noticed? ____________________________________________

3.4. Have you ever been for an examination and/or treatment for the abovementioned complaints?

yes  no

3.5. Have you had an infection/cold in the last 2 weeks?

yes, without fever  yes, with fever  no

**4. Autonomic anamnesis**

**Appetite**  normal  reduced  increased  cravings

**Diet**  mixed diet  ovo-lacto vegetarian^^[[1]](#footnote-2)^^  vegetarian  vegan
(several possible)

Other diet  yes  no If yes, which? __________________________

How often do you eat fruit? _______times/day

How often do you eat vegetables? _______times/day

How often do you eat whole-meal products (e.g., bread, pasta, cereali)? _______/day

**Weight**  constant  weight loss*  weight gain*

*of _____kg in _____ months

**Bowel movement**  normal  diarrhea  constipation  blood in stool

black or tarry stools

**Urination**  normal  at night If at night, how often? ______________

**Nicotine**  yes  no  in the past

If yes: ____ cigarettes/day

If in the past: ____ cigarettes/day, over what period/until when: ________________

**Alcohol**  never  rarely  occasionally  regularly

__________number of drinks/week

**Sleep**  good  insomnia  sleep disorders

snoring

Average sleep duration: _____ hours/day

**Allergies**  no  yes If so, which? _________________________________

**For women:**

**Menstruation** for the first time at the age of: _____ regularly:  yes  no  menopause

__________________________________________________________________________________________

**Medication**  no  yes

If yes, which medication (name) and dosage (morning, midday, evening)?

| Medication (name) | Dose | Morning | Noon | Evening |
| --- | --- | --- | --- | --- |
|  |  |  |  |  |
|  |  |  |  |  |
|  |  |  |  |  |
|  |  |  |  |  |
|  |  |  |  |  |
|  |  |  |  |  |

**Food supplements**  no  yes If yes, which? __________________________

**5. Known pre-existing illnesses/diagnoses**

|  | No | Yes | (Since) when? | Which diagnoses? |
| --- | --- | --- | --- | --- |
| Heart diseases |  |  |  |  |
| Vascular diseases  (e.g., stroke, thrombosis) |  |  |  |  |
| Lung diseases  (e.g., asthma, COPD, pulmonary embolism) |  |  |  |  |
| High blood pressure |  |  |  |  |
| Cancer |  |  |  |  |
| Diabetes (diabetes mellitus) |  |  |  | If yes,  Type 1  Type 2 |
| Elevated blood lipid levels  (e.g. cholesterol, neutral fats) |  |  |  |  |
| Thyroid disease |  |  |  |  |
| Other |  |  |  |  |

**6. Diseases in the family**

|  | No | Yes | Which (and for whom in the family; age of onset)? |
| --- | --- | --- | --- |
| Heart attack – before the age of 60 |  |  |  |
| – after the age of 60 |  |  |  |
| Sudden cardiac death |  |  |  |
| High blood pressure |  |  |  |
| Other heart disease |  |  |  |
| Vascular diseases  (e.g., stroke, thrombosis) |  |  |  |
| Diabetes (diabetes mellitus) |  |  | if yes  Type 1  Type 2 |
| Cancer diseases  (e.g., bowel and breast cancer) |  |  |  |
| Lung diseases  (e.g., asthma, COPD) |  |  |  |
| Increase in blood lipids  (e.g. cholesterol, neutral fats) |  |  |  |
| Other diseases  (e.g., thyroid gland) |  |  |  |
| Epilepsy/seizure disorders |  |  |  |
| Repeated loss of consciousness |  |  |  |
| Marfan syndrome |  |  |  |
| Other |  |  |  |

**Musculoskeletal system**

6.1. Do you currently have complaints or an injury in the musculoskeletal system?

no  yes

6.2. Have there been any previous complaints or injuries to the musculoskeletal system (e.g., broken bones) that have restricted your physical activity?

no  yes

6.3. Have medical or physiotherapeutic treatments (including aids) been prescribed to treat strains that restrict physical activity?

no  yes

6.4. Have surgical procedures been performed on the musculoskeletal system (e.g., on the meniscus)?

no  yes

**7. Preventive medical check-ups**

|  | Last (month/year) | Abnormal findings (yes/no)  If yes, diagnosis? |
| --- | --- | --- |
| Colonoscopy/stool blood test |  |  |
| For women: Gynecology |  |  |
| For men: Urology/family doctor or general practitioner |  |  |
| Skin cancer screening |  |  |
| Dentist |  |  |
| Vaccination status |  |  |

**8. Prevention programs**

8.1 Do you participate in exercise/prevention programs?

no  yes, which/what?

8.2 Do you conduct prevention training (e.g., independent training to prevent injuries, FIFA11+)?

no  yes, which/what?

**9. Social anamnesis**

Shift work  no  yes

Weekly working hours ________ hours/week

Caring responsibilities (e.g., children, nursing care)  no  yes

Signature (person examined): _______________________________________

**Examination form**

**Date: Name: Date of birth:**

**Anthropometric Data**

| **Height (cm): ______** | **Weight (kg): _____** | **BMI (kg/m^2^): _____** | **Abdominal circumference (cm): _____** | **Waist-to-height-ratio: ______** |
| --- | --- | --- | --- | --- |
| **Optional: Body composition**  Bioelectrical impedance analysis: muscle mass (____%, ____ kg), fat mass (___ %, ____ kg)  Measurement of skin fold thickness: ___%body fat  Other | | | | |
| **Observations** | | | | |

| **Heart rate** (S/min): ______ **Blood pressure** (mmHG): ____/____ (____/____)______ |
| --- |
| **Observations** |

**Physical examination**

|  | | **Observations** | | |
| --- | --- | --- | --- | --- |
|  | | **-** | **+** | **Documentation** |
| **External appearance**  e.g., Marfan stigmata (kyphoscoliosis, high arched palate, pectus excavatum, arachnodactyly, hyperlaxity, or others) | |  |  |  |
| **Skin**  e.g., herpes simplex virus, lesions indicating tinea corporis, edema, congestive dermatosis | |  |  |  |
| **Eyes, Ears, Nose, Neck** | View:  R 20/  L 20/  Corrected?  yes  no |  |  |  |
| **Lymph node status** |  |  |  |  |
| **Heart** | e.g., auscultation while standing, auscultation while lying down, Valsalva maneuver |  |  |  |
| **Peripheral vascular status**  e.g., radial, inguinal, foot pulses  varicosis | |  |  |  |
| **Lungs**  Inspection, palpation, percussion, auscultation | |  |  |  |
| **Abdomen**  Inspection, palpation, percussion, auscultation | |  |  |  |
| **Neurological**  e.g., sensitivity, peripheral reflex status | |  |  |  |

**Examination of the musculoskeletal system**

|  | | **Observations** | | |
| --- | --- | --- | --- | --- |
| **Localization** | | **~~-~~** | **+** | **Documentation** |
| **Head/neck** | |  |  |  |
| **Torso/thorax** | |  |  |  |
| **Upper extremity** | **Shoulder** |  |  |  |
|  | **Upper arm/Elbow** |  |  |  |
|  | **Forearm/**  **Hand joint** |  |  |  |
|  | **Hand** |  |  |  |
|  | **Muscles/ Tendons** |  |  |  |
| **Spine** | **Cervical spine** |  |  |  |
|  | **Thoracic spine** |  |  |  |
|  | **Lumbar spine** |  |  |  |
|  | **Sacrum/ Coccyx/ Sacroiliac joint** |  |  |  |
| **Lower extremity** | **Pelvis/**  **Groin** |  |  |  |
|  | **Hip/**  **Thigh** |  |  |  |
|  | **Knee** |  |  |  |
|  | **Lower leg, Upper ankle joint** |  |  |  |
|  | **Foot** |  |  |  |
|  | **Muscles/ Tendons** |  |  |  |

1. Consumption of eggs and dairy products but not fish and meat. [↑](#footnote-ref-2)
